# A metagenomic analysis of the camel rumen’s microbiome identifies the major microbes responsible for lignocellulose degradation and fermentation

**DOI:** 10.1186/s13068-018-1214-9

**Published:** 2018-08-02

**Authors:** Javad Gharechahi, Ghasem Hosseini Salekdeh

**Affiliations:** 1 Human Genetics Research Center, Baqiyatallah University of Medical Sciences, Tehran, Iran; 2 Department of Systems Biology, Agricultural Biotechnology Research Institute of Iran, Agricultural Research Education, and Extension Organization, Karaj, Iran; 3 Department of Molecular Sciences, Macquarie University, Sydney, NSW Australia

**Keywords:** Camel, Rumen metagenome, Microbiome, Binning, Carbohydrate active enzymes

## Abstract

**Background:**

The diverse microbiome present in the rumen of ruminant animals facilitates the digestion of plant-based fiber. In this study, a shotgun metagenomic analysis of the microbes adhering to plant fiber in the camel rumen was undertaken to identify the key species contributing to lignocellulose degradation and short chain volatile fatty acids (VFA) fermentation.

**Results:**

The density of genes in the metagenome encoding glycoside hydrolases was estimated to be 25 per Mbp of assembled DNA, which is significantly greater than what has been reported in other sourced metagenomes, including cow rumen. There was also a substantial representation of sequences encoding scaffoldins, dockerins and cohesins, indicating the potential for cellulosome-mediated lignocellulose degradation. Binning of the assembled metagenome has enabled the definition of 65 high-quality genome bins which showed high diversity for lignocellulose degrading enzymes. Species associated to *Bacteroidetes* showed a high proportion of genes for debranching and oligosaccharide degrading enzymes, while those belonging to *Firmicutes* and *Fibrobacteres* were rich in cellulases and hemicellulases and thus these lineages were probably the key for ensuring the degradation of lignocellulose. The presence of many “polysaccharide utilization loci” (PULs) in *Bacteroidetes* genomes indicates their broad substrate specificity and high potential carbohydrate degradation ability. An analysis of VFA biosynthesis pathways showed that genes required for the synthesis of acetate were present in a range of species, except for *Elusimicrobiota* and *Euryarchaeota*. The production of propionate, exclusively via the succinate pathway, was carried out by species belonging to the phyla *Bacteroidetes*, *Firmicutes*, *Spirochaetes* and *Fibrobacteres*. Butyrate was generated via the butyrylCoA: acetate CoA-transferase pathway by *Bacteroidetes* and *Lentisphaerae* species, but generally via the butyrate kinase pathway by *Firmicutes* species.

**Conclusion:**

The analysis confirmed the camel rumen’s microbiome as a dense and yet largely untapped source of enzymes with the potential to be used in a range of biotechnological processes including biofuel, fine chemicals and food processing industries.

**Electronic supplementary material:**

The online version of this article (10.1186/s13068-018-1214-9) contains supplementary material, which is available to authorized users.

## Background

The animal gastrointestinal tract supports a diverse community of bacteria, protozoa, fungi and archaea [1]. These symbiotic organisms contribute to the nutrition of the host animal by converting non-digestible feedstuff into readily absorbable compounds, and are thought to contribute to the host’s physiology and health [2]. This symbiosis is particularly important for herbivorous animals, which are unable to endogenously synthesize the hydrolytic enzymes required for the degradation of the plant lignocellulosic material that form a major component of their diet. During fetal development, the host’s gastrointestinal tract undergoes anatomical and physiological changes to allow it to support the symbionts; these include the formation of an enlarged pre-gastric (the rumen of foregut fermenters) or post-gastric (the caecum and/or colon of hindgut fermenters) fermentation chamber [3]. The resulting boost in the ability to retain ingested material, along with a near neutral pH is conducive for microbial colonization in these specialized organs. Due to the relatively acidic pH and rapid transition of digesta through the midgut or small intestine, these organs tend to be less populated by microbes [3].

The ruminant animal’s stomach comprises four distinct compartments, namely the rumen, reticulum, omasum and abomasum. The anaerobic rumen, which accounts for as much as 55% of the volume of an adult ruminant’s stomach [4] is the main site of the microbial fermentation of plant lignocellulosic material. The function of the downstream compartment, omasum, is to enhance the effectiveness of the time-consuming fermentation process [5]. Plant lignocellulose is degraded in the rumen into monomers and oligomers which are further converted into short chain volatile fatty acids (VFAs), a process associated with the production of acetate, propionate and butyrate, along with carbon dioxide, hydrogen and methane [3]. The VFAs serve as a major source of energy for the host because they are easily absorbed into the blood, which ferries them to the liver where gluconeogenesis takes place [6, 7]. The rumen microbes also facilitate the absorption of minerals and water, provide vitamin B and contribute to urea recycling [3]. Their entry, along with the rumen digesta, into the abomasum exposes them to HCl and to the action of various host digestive enzymes, thereby serving as a source of proteins and amino acids for the host.

The rumen’s microbiome is dominated by obligate anaerobic microorganisms originating from all three taxonomic domains of life, i.e., Archaea, Bacteria, and Eukarya. Bacteria are the most abundant group accounting for more than 95% of microbial biomass of the rumen and they make the greatest contribution towards the breakdown and fermentation of plant feedstuffs. Conventional culture-based methods have been used to identify at least 200 bacterial species adapted to the conditions in the rumen [8]. With the application of next-generation sequencing (NGS) technologies for studying microbes inhabiting the rumen, our understanding of rumen microbial diversity and function has been significantly increased. NGS-based metagenomics surveys were first oriented to the taxonomic and phylogenetic analysis of microbial communities found in feces and rumen content of diverse animal species [4, 9–14]. However, they were soon adapted to characterize the constituents of the rumen microbiome, a knowledge which could be exploited for the isolation and characterization of enzymes or pathways of industrial or biotechnological value. The approach has been widely applied to understand the nature of the carbohydrate degradation processes carried out in both ruminant and non-ruminant animals [15–20], as well as in engineered environments including anaerobic digesters and biogas fermenters [21–25].

Advances in bioinformatics have now enabled us to reconstitute complete or draft genomes from metagenomic sequences, an achievement that allows us to get insight into the microbiome’s metabolic networks, functional capabilities and species interactions [23, 26, 27]. Recently, Stewart, et al. [20] have assembled 913 microbial draft genomes based on 800 Gbp of metagenomic sequence obtained from the bovine rumen, identifying a number of previously uncharacterized bacterial strains and species. A remaining challenge relates to the problem of obtaining a complete, or at least a near complete genome sequence for low abundance species [26]. Among the approaches suggested to address this challenge are to increase sequencing depth and to exploit the capacity to acquire sequence from single cells [26, 28].

Recent analysis of rumen microbiota from 32 animal species showed that the composition of the rumen microbiota is largely determined by diet and it is likely less influenced by the host [29]. The camel diet is dominated by a variety of woody shrubs and tree biomasses, along with various halophytes, species which are not favored by most ruminants [10, 30]. Consequently, their rumen microbes must, therefore, have the capacity to degrade such recalcitrant feedstocks which are rich in lignocellulosic materials. In a recent study, 16S rRNA sequencing was used to identify the species composition of the camel’s rumen microbiome [10]. Here, partial and near complete genome sequences of 64 bacterial and one archaeal species have been presented, along with an analysis of the species’ potential for lignocellulose degradation and VFA fermentation. A detailed profiling of the camel rumen’s carbohydrate-active enzymes (CAZymes) is given, along with a taxonomic treatment of its fiber-adherent microbiome.

## Methods

### Sampling of rumen digesta and DNA extraction

Rumen samples were collected from three healthy adult 2–5-year-old camels. The sampling procedure has been described elsewhere [10]. Genomic DNA was extracted using a QiaAmp^®^ DNA Stool Mini Kit (Qiagen, Valencia, CA, USA) according to the manufacturer’s protocol for isolation of DNA from stools for pathogen detection. The quality and the quantity of DNA extracted were determined using both NanoDrop spectrophotometry and gel electrophoresis.

### Metagenome library preparation and sequencing

Metagenome DNA sequencing was performed at the Beijing Genome Institute (BGI, Shenzhen, China) according to the standard protocols. A library was prepared using a Nextera DNA Library Preparation Kit (Illumina, San Diego, CA, USA), according to the manufacturer’s protocol. Briefly, 200 µg metagenomic DNA was sheared using Covaris (Covaris Inc, Massachusetts, USA), and DNA fragments were end-repaired, adenylated, and ligated with Illumina sequencing adaptors. DNA fragments with mean size of 350 nt were purified based on bead-size selection using the Agencourt AMPure XP beads (Beckman Coulter, Beverly, MA, USA). After PCR amplification, the library was sequenced in paired-end mode (2 × 90 bp) on a single lane of an Illumina Hiseq2000 system. The number of clean reads acquired was 263 million, equivalent to 23 Gbp.

### De novo assembly

High-quality reads were assembled de novo into contigs using IDBA-UD v1.1 and Spades v3.10 software [31, 32]. IDBA-UD was used for an initial assembly using the following parameters: –mink 20, –maxk 80, –step 10, and –min_contig 200. Spades was subsequently used for the final assembly using k-mers 25,35,45,55,65,75,83. The contigs generated by the IDBA-UD program were included in the final assembly using flag –trusted-contigs. Combining IDBA-UD and Spades resulted in a better assembly with higher assembly statistics. To determine the percentage of assembled reads and to obtain coverage profiles of scaffolds, paired-end reads were mapped to scaffolds using BBMap (version 36.92) with default parameters. Only scaffolds longer than 200 nt were retained. The assembled scaffolds have been submitted to the Integrated Microbial Genomes database (IMG) under submission ID 142919 and analysis project Ga0206072.

### Gene discovery and metagenome annotation

Prodigal v2.6.3 software [33], operating in metagenome mode, was used to identify open reading frames (ORFs). Predicted translation products were functionally annotated against the cluster of orthologous group genes (COGs), Pfam domains, SEED subsystem, and the Kyoto Encyclopedia Genes and Genomes (KEGG) pathways using COGNIZER software [34]. Protein sequences were screened for candidate CAZymes against a set of hidden markov model (HMM) profiles representing a range of enzyme families, including glycoside hydrolases (GHs), carbohydrate esterases (CEs), polysaccharide lyases (PLs), cohesion, dockerin, carbohydrate binding modules (CBMs), and auxiliary activity enzymes (AAs) [35]. To identify potential polysaccharide-utilization loci (PULs), two additional HMM profiles (representing SusC and SusD) were included. HMM profiles were retrieved from either dbCAN v5 or Pfam v31 [35, 36]. The search was accomplished using hmmscan implemented in HMMER v3.1b2, with a query coverage 30% and an *e*-value cutoff 1e−5 for alignments longer than 80 residues, and 1e−3 for those shorter than this threshold [37]. To exclude overlaps, hits obtained were sorted on the basis of their *e*-value, retaining only the one associated with the lower *e*-value.

A search using Jackhammer software, based on a cutoff score of 700, was used to identify potential cellulosomal scaffodins, as described elsewhere [22]. The templates used for an iterative similarity search were the scaffoldins CbpA (AAA23218.1, *Clostridium cellulovorans*), ScaB (AAT79550.1 *Bacteroides cellulosolvens*), CipA (Q06851, *Clostridium thermocellum*), CipC (AAC28899.2, *Clostridium cellulolyticum* H10), ScaB (CAC34385.1, *Ruminococcus flavefaciens* 17), CipA (BAA32429.1, *Clostridium josui*), CipA (AAK78886.1, *Clostridium acetobutylicum* ATCC 824), ScaA (AAG01230.2, *Pseudobacteroides cellulosolvens*). The peptide sequences of predicted GH enzymes were subjected to a BlastP search against the NCBI non-redundant protein database (ftp://ftp.ncbi.nlm.nih.gov/blast/db/FASTA/nr.gz, March 2017) using an *e*-value cutoff of 1e−3 and a maximum target sequence of 20. The resulting outputs were loaded into MEGAN6 software to taxonomically identify the source of each putative GH enzyme, based on the lowest common ancestor (LCA) algorithm [38].

The CAZyme profile of the camel rumen metagenome was compared with those present in both the cow and the moose rumen [16, 39], in biogas reactors [21, 22], and in elephant feces [15]. Contigs longer than 1000 nt were subjected to ORF prediction and a dbCAN database search. For the identification of over- or under-represented CAZyme families, Fisher’s exact test was applied and *p* values were corrected for multiple testing using the false discovery rate (FDR) method, as implemented in the R package fisher.test for count data v3.4.2.

### Community profiling

The community structure of the camel rumen’s microbiome was explored using 16S rRNA amplicon pyrosequencing [10]. The program CommunityM (https://github.com/dparks1134/CommunityM.git) was used to identify 16S rRNA coding sequence in the assembled scaffolds. The retrieved sequences were assigned to taxon using an RDP classifier, applying a confidence threshold of 0.8 and a sequence similarity level of 97% [40].

### Metagenome binning and population genome bin recovery

To reconstruct population genome bins (GBs), scaffolds longer than 2500 nt were clustered based on their coverage and tetranucleotide frequency, using MetaBat v0.32.4 software running in ensemble (-B 20) and superspecific modes [41]. GBs were assessed in terms of their completeness, contamination and strain heterogeneity using the Checkm program, which employs a set of 43 clade-specific single copy marker genes [42]. Only those GBs assessed as being > 50% complete and < 10% contaminated were retained. Contigs in each GB were used to recruit unassembled sequences and to further extend their length using PRICE v1.2 software [43] run in target mode, with the parameters -fpp reads_R1.fq reads_R2.fq 350 95 -icf bin.fa 1 1 5 –nc 10 –dbmax 72 –mol 30 –tol 20 -mpi 90 –target 90 0. GBs were assigned to taxon using the Phylophlan program, which uses > 400 universally conserved proteins encoded by 3171 genomes [44]. The relative abundance of individual taxa was measured by mapping the clean reads against the binned scaffolds, after a normalization step based on the size of the relevant GB. The annotation of the gene content of each GB was performed using PROKKA v1.12 software [45]. CAZymes encoded within each GB were identified as described above for the assembled scaffolds.

## Results

### Metagenome sequencing and assembly

The sequencing yielded 263,160,260 high-quality paired-end reads, representing 23 Gbp of sequences. A de novo assembly using a combination of IDBA-UD and Spades software resulted in the recognition of 1,502,637 contigs of length > 200 nt and having an N50 of 2914 nt. The length of the longest scaffold was 412,605 nt, with 21,322 scaffolds longer than 10,000 nt. Back alignment of the reads against the assembled scaffolds indicated that 75% of them were incorporated within an assembly, giving a mean coverage of 10.8×. Based on the presence of predicted open reading frames, some 2,736,491 protein encoding genes of mean length 586.2 nt were identified; of these, 56% (1,536,919, with a mean length of 722 nt) were predicted to represent full length genes. A total of 1,641,131 of the sequences matched entries in the COG database (59.9%), 1,974,145 did so in the KEGG database (72.1%), 1,731,303 in the Pfam database (63.3%) and 541,522 in the SEED subsystem database (19.8%). The COG-based classification suggested that 12.9% of the proteins could only be given a general functional prediction (e.g., biochemical activity), while 9.2% were associated with carbohydrate transport and metabolism, 8.4% with amino acid transport and metabolism, 8.3% with DNA replication, recombination and repair, 7.5% with cell wall membrane biogenesis, 7.5% with translation, ribosomal structure and biogenesis, 5.6% with transcription, 4.9% with energy production and conversion and 4.4% with signal transduction.

### Analysis of taxonomical assignments of the camel rumen’s microbiome

An inspection of the 16S rRNA gene sequences recovered from the assembled scaffolds revealed that the microbial community was dominated by species belonging to the bacterial phyla *Bacteroidetes* (35.4%) and *Firmicutes* (27.4%); less well represented were the phyla *Spirochaetes* (6.6%), *Proteobacteria* (4.1%), *Verrucomicrobia* (1.4%), *Tenericutes* (1.0%), SR1 (0.8%), *Fibrobacteres* (0.5%), *Lentisphaerae* (0.3%), *Synergistetes* (0.3%) and *Elusimicrobia* (0.3%). Under 1% of the sequences were derived from species belonging to the archaea, indicating their relatively lower representation in the camel rumen compared to bacterial sequences. The species assignment of around 21% of the sequences could not be allocated. There was some inconsistency between this metagenome-based assessment of the community structure and the one derived from amplicon-based profiles [10]; the latter method over-estimated the relative abundance of *Bacteroidetes*, *Firmicutes* and *Fibrobacteres* species by around 10, 6 and 4%, respectively, perhaps due to some bias associated with the PCR amplification. The metagenome data revealed a somewhat greater representation of *Proteobacteria* species than was suggested by the 16S-rRNA amplicon pyrosequencing data (4.1% vs 2.6%).

### The identification of genome bins from the metagenome sequences

Scaffolds of length > 2500 nt were clustered on the basis of their tetranucleotide frequencies and their coverage profiles, resulting in the identification of 65 genome bins associated with a completeness score of > 70% and a < 10% level of contamination (Table 1); 35 of the bins were associated with a completeness score of > 90%, and 57 with a score of > 80%. A set of 12 bins was associated with a completeness score of > 80%, but their contamination level was 10–30%; as a result these were excluded from the subsequent analysis. The size of the bins which were retained ranged from 0.98 × 10^6^ to 4.08 × 10^6^ bp, and their GC content varied from 33 to 67%.

**Table 1:** Basic genome characteristics of the recovered genome bins (GBs)

BinID	Genome size (Mbp)	GC content	No. contigs	No. GHs	No. CBMs	No. dockerin	No. cohesin	SLH domain	Taxonomic label (phylum)	Com^a^	Con^b^	Het^c^	Abu^d^
Bin48	4.08	38.53	270	38	16	0	0	0	Firmicutes	72.9	7.3	2.17	0.2
Bin49	3.6	52.96	125	176	33	11	0	0	Bacteroidetes	91.5	3.1	16.67	0.4
Bin53	3.59	42.52	93	61	16	1	0	0	Bacteroidetes	92.1	4.48	31.25	0.17
Bin55	3.34	49.81	53	70	6	0	0	0	Firmicutes	98.97	3.26	0.0	0.12
Bin58	3.48	48.22	132	166	29	11	0	0	Bacteroidetes	94.82	5.33	32	0.47
Bin60	3.18	50.68	199	51	7	0	1	0	Firmicutes	91.2	9.86	0.0	0.09
Bin61	3.16	44.99	168	82	6	0	0	0	Firmicutes	91.6	9.42	26.3	0.12
Bin62	3.15	47.7	70	102	16	1	0	0	Bacteroidetes	93.90	0.68	33.3	0.38
Bin63	3.13	47.02	85	54	9	0	0	0	Bacteroidetes	95.05	7.86	45	0.13
Bin64	3.2	54.78	127	90	19	0	0	0	Fibrobacteres	87.6	5.13	40	0.37
Bin65	3.02	48.36	104	132	8	0	0	0	Bacteroidetes	91.1	4.44	47.06	0.44
Bin68	3	43.04	181	25	29	0	0	0	Bacteroidetes	97.58	6.72	23.08	0.1
Bin69	3.1	42.78	57	66	13	0	0	0	Firmicutes	91.34	2.58	23.08	0.23
Bin70	2.98	42.76	165	60	1	0	0	1	Firmicutes	90.29	5.96	0.0	0.1
Bin73	2.9	48.96	51	34	63	0	1	0	Firmicutes	98.07	0.0	0.0	0.14
Bin75	2.9	54.38	88	155	36	0	0	0	Bacteroidetes	86.19	3.37	0.0	0.23
Bin81	2.84	49.39	149	67	28	21	4	0	Firmicutes	86.01	6.1	50	0.11
Bin82	2.97	50.1	88	118	11	3	0	0	Bacteroidetes	85.57	3.58	66.67	0.7
Bin83	2.88	46.06	56	47	4	0	0	0	Firmicutes	94.68	1.42	0.0	0.37
Bin86	2.75	44.2	101	73	4	5	0	0	Bacteroidetes	93.39	2.43	83.3	0.24
Bin87	2.67	50.13	143	159	23	0	0	0	Bacteroidetes	84.23	5.95	4.55	0.2
Bin88	2.63	53.33	82	96	36	0	0	0	Firmicutes	96.95	0.23	100	0.1
Bin91	2.6	56.08	144	83	3	0	0	0	Firmicutes	88.34	8.56	0.0	0.1
Bin93	2.6	50.99	93	63	15	0	0	0	Fibrobacteres	97	1.1	0.0	0.2
Bin94	2.59	36.01	100	37	4	1	1	0	Firmicutes	99.16	7.38	41.18	0.12
Bin96	2.57	43.38	53	68	5	0	0	0	Firmicutes	95.77	0.53	50	0.3
Bin97	2.55	43.25	358	74	8	0	0	0	Firmicutes	83.12	4.19	11.76	0.09
Bin98	2.54	67.77	178	21	11	0	0	0	Lentisphaerae	79.22	2.36	0.0	0.23
Bin99	2.51	52.29	102	171	15	0	0	0	Bacteroidetes	94.29	1.43	33.33	0.35
Bin100	2.51	47.13	199	42	5	0	0	78	Firmicutes	84.29	3.25	12.5	0.09
Bin101	2.49	45.35	104	43	7	0	0	0	Spirochaetes	93.01	0.12	0.0	0.09
Bin103	2.48	43.12	227	39	9	0	0	0	Spirochaetes	75.12	1.4	33.33	0.06
Bin104	2.48	66.95	126	20	9	0	0	0	Lentisphaerae	87.17	4.08	0.0	0.12
Bin105	2.47	50.54	64	40	3	0	1	0	Firmicutes	93.35	1.58	0.0	0.09
Bin106	2.47	44.91	320	65	9	0	1	0	Firmicutes	87.75	6.81	33.33	0.1
Bin108	2.45	56.29	94	69	8	0	0	0	Bacteroidetes	87.84	6.06	35	0.29
Bin110	2.32	38.96	79	36	9	0	0	0	Firmicutes	92.16	7.9	1.89	0.11
Bin112	2.3	56.63	54	108	3	1	0	0	Bacteroidetes	92.92	0.95	0.0	0.5
Bin113	2.3	38.95	325	60	6	0	1	0	Firmicutes	78.15	6.32	20	0.08
Bin114	2.3	50.06	47	20	9	0	0	0	Bacteroidetes	87.37	2.8	41.67	0.18
Bin115	2.27	49.96	85	39	5	0	0	0	Firmicutes	97	7.62	21.43	0.2
Bin117	2.2	47.78	238	74	4	0	0	0	Firmicutes	89.85	5.53	14.29	0.13
Bin119	2.18	54.56	77	93	3	0	0	0	Bacteroidetes	95.38	1.52	25	0.42
Bin121	2.19	43.55	76	82	7	2	0	0	Bacteroidetes	70.16	7.76	40	0.2
Bin122	2.14	52.71	54	94	3	1	0	0	Bacteroidetes	95.08	0.19	0.0	0.3
Bin123	2.27	54.48	91	100	12	1	0	0	Bacteroidetes	91.19	4.05	36.36	0.62
Bin124	2.1	46.44	33	26	8	0	0	0	Bacteroidetes	92.1	0.81	0.0	0.22
Bin128	2.08	51.55	249	19	2	0	0	9	Firmicutes	86.78	4.08	30	0.09
Bin129	2.07	50.58	165	82	6	0	2	0	Bacteroidetes	84.21	2.7	54.55	0.29
Bin131	2.16	44.49	52	93	6	0	0	0	Bacteroidetes	79.7	1.98	50	0.53
Bin133	2.07	55.82	50	83	6	0	0	0	Bacteroidetes	87.62	0.95	100	0.51
Bin134	2.03	49.33	60	25	11	0	0	0	Bacteroidetes	90.14	3.15	76.19	0.17
Bin136	2.01	49.87	42	54	16	5	1	0	Bacteroidetes	75.56	2.79	43.75	0.25
Bin138	2.09	49.53	31	51	12	0	0	0	Bacteroidetes	94.29	0.66	0.0	0.45
Bin140	1.96	55.28	33	97	7	0	0	0	Bacteroidetes	93.4	2.06	0.0	0.36
Bin142	2.15	55.36	80	97	11	0	0	0	Bacteroidetes	89.52	0.48	0.0	0.81
Bin143	1.94	48.69	55	14	7	0	0	0	Bacteroidetes	92.74	0.0	0.0	0.29
Bin145	2.02	50.62	105	26	24	0	0	0	Bacteroidetes	93.2	3.4	0.0	0.26
Bin147	1.83	50.55	205	61	10	0	0	0	Bacteroidetes	85.77	0.71	0.0	0.27
Bin154	1.76	48.53	183	14	5	0	0	0	Bacteroidetes	83.69	5.1	0.0	0.13
Bin164	1.58	33.65	25	10	4	0	0	0	Tenericutes	97.33	1.33	0.0	0.16
Bin165	1.56	48.77	153	22	3	0	0	1	Firmicutes	82.89	4.26	0.0	0.07
Bin174	1.35	48.57	147	8	4	0	0	0	Spirochaetes	90.8	4.6	40	0.12
Bin187	1.2	55.2	45	1	2	2	0	0	Euryarchaeota	80.65	0.81	0.0	0.15
Bin206	0.98	37.13	144	4	2	1	0	0	Elusimicrobia	83.6	4.25	0.0	0.08

GBs were taxonomy assigned at the phylum level using universally conserved marker proteins using CheckM

^a^Compeletness, ^b^ contamination, ^c^ strain heterogeneity, ^d^ abundance

Taxonomic identification based on the presence of > 400 conserved marker genes within the bins was largely consistent with the conclusions based on the 16S rRNA gene sequences. Thus, 32 of the bins identified species belonging to the phylum *Bacteroidetes*, order *Bacteroidales* and four to the genus *Prevotella*. The second most abundant group (23 bins) identified species belonging to the phylum *Firmicutes*, order *Clostridiales*. The remaining nine bins were allocated to the phyla *Spirochaetes* (three), *Fibrobacteres* (two), *Lentisphaerae* (two), *Tenericutes* (one) and *Elusimicrobia* (one). Bin #187 identified an Archaea species belonging to the phylum *Euryarchaeota*. Only two of the bins (#64 and #93) were assignable to the species level, both identifying *Fibrobacter succinogenes*, representing known members of the camel rumen’s microbiome. No bins corresponding to *Proteobacteria* species were recovered, even though the 16S rRNA-based analysis had suggested that these species accounted for > 4% of the rumen community.

By mapping reads against the binned scaffolds, it was possible to show that the bins associated with species belonging to the phylum *Bacteroidetes* were the most well represented members of the rumen’s microbiome (0.10–0.80%), followed by *Firmicutes* (0.07–0.37%). The 17 bins present at an abundance of < 0.1% were assumed to represent species present at a low frequency. In contrast, the two relatively high-frequency *Fibrobacteres* bins #93 and #64 represented, respectively, 0.20 and 0.37% of the metagenomic sequence, consistent with the high frequency associated with species belonging to this phylum predicted by 16S rDNA analysis. Bin #164, which occurred at a frequency of 0.16%, was the only representative of the phylum *Tenericutes*.

### The carbohydrate-active enzyme repertoire of the camel rumen’s microbiome

The capacity of the camel’s rumen microbiome to convert lignocellulosic materials into VFAs depends on their genomic constituent for carbohydrate-active enzymes (CAZymes). A scan of the set of ORFs uncovered by the metagenome sequencing revealed 98,206 relevant sequences (3.6% of the overall set of open reading frames): of these 40,555 (41.0%) encoded a GH, 12,603 (12.8%) a CBM, 2102 (2.1%) a cellulosome binding domain (1820 dockerin and 282 cohesin domains), 2437 (2.5%) a PL, 23,418 (23.8%) a GT, 14,564 (14.8%) a CE, 1092 (1.1%) an S-layer homology domain (SLH) and 1434 (1.5%) a AA. Among the predicted CAZyme-encoding sequences, 10,809 contained two or more distinct CAZy domains.

### GHs

The set of > 40,000 encoded GH enzymes fell into 104 families. The most prevalent of these was GH43 (3641 sequences), while the 11 families GH2, GH3, GH5, GH13, GH23, GH25, GH28, GH31, GH43, GH78 and GH109 constituted > 50% (20,992) of the sequences. The putative GHs were classified according to both their main substrate and their mode of action into endoglucanases, endohemicellulases, debranching enzymes and oligosaccharide degrading enzymes. The set of endo and exoglucanases included endo-β-1,4-glucanases, glucan β-1,3-glucosidases and cellobiohydrolases were dominated by the families GH5 (1724 genes), GH9 (738 genes), GH44 (22 genes), GH45 (32 genes) and GH48 (four genes). The endohemicellulases (endo-β-1,4-xylanases, endo-β-1,3-xylanases, β-mannanases and polygalacturonases) were represented by 3671 sequences distributed among the seven families GH8, GH10, GH11, GH12, GH26, GH28 and GH53. Within this group, polygalacturonases belonging to GH28 were encoded by 1304 sequences, while the GH10 and GH11 xylanase members were represented by, respectively, 858 and 122 sequences. Genes encoding enzymes involved in the degradation of carbohydrate side chains were also strongly represented: these included members of GH51 (α-l-arabinofuranosidases, 846 genes), GH54 (α-l-arabinofuranosidases, 31 genes), GH67 (α-glucuronidases, 176 genes) and GH78 (α-l-rhamnosidases, 1002 genes). The 11,225 genes (26.8% of all GHs) encoding oligosaccharide degrading enzymes (β-glucosidase, β-galactosidase, exo-β-1,4-glucanase and xylan 1,4-β-xylosidase) were represented by members of GH1, GH2, GH3, GH29, GH35, GH38, GH39, GH42, GH43 and GH9. Within this group, members of GH2, GH3 and GH43 accounted for 80% of the full set.

A comparison of GH frequencies predicted in contigs of minimum length 1000 nt with those present in the bovine and moose rumens [16, 39], in an agricultural biogas fermenter [22], in elephant feces [15] and in an anaerobic digester [21] is given in Additional file 2: Table S1. The analysis suggested that the camel rumen microbiome encoded 25.3 GHs per Mbp of assembled DNA, a density comparable to that of both the moose rumen (23.8) and in elephant feces (22.9), but rather more than in the bovine rumen (10.1), the biogas fermenter (16.4) and the anaerobic digester (15.3) (Table 2).The metagenome most similar to that of the camel rumen in terms of GHs was that of the moose, as the only GHs which were more frequent in the camel’s belonged to either GH9 or GH39 (FDR_adjusted *p* value < 0.05) (Additional file 2: Table S1). The elephant feces metagenome showed a degree of similarity, with just 26 of the 114 GH families represented differing in frequency (Additional file 2: Table S1).

**Table 2:** Comparing assembly statistic and hydrolytic potential of the camel rumen’s metagenome with that of the bovine and the moose rumen, elephant feces, and biogas reactors

Metagenome	Metagenome size (Gbp)	Assembled DNA (Gbp)	No. contigs	No. ORFs	No. GHs	No. CBMs	No. CEs	No. PLs	GHs/Mbp	%GH	Refs.
Camel rumen	23	1.26	*333,382*	1,342,629	*31,832*	6920	*11,308*	*1875*	*25.3*	2.37	This study
Bovine rumen	*111.4*	*1.92*	179,088	*1,744,737*	19,465	*7198*	7740	742	10.13	1.11	Hess et al. [16]
Anaerobic digester	51	0.51	164,127	592,362	7816	2382	2775	264	15.3	1.32	Campanaro et al. [21]
Biogas reactor	58.7	0.84	236,489	930,135	13,787	4530	4934	677	16.4	1.48	Gullert et al. [22]
Elephant feces	54.7	0.93	260,535	1,005,764	21,348	4449	7097	897	22.9	2.12	Ilmberger et al. [15]
Moose rumen	–	0.262	26,172	235,465	6247	1301	1846	476	23.8	*2.65*	Svartström et al. [39]

Only contigs larger than 1 Kbp were included

For easy tracking the extreme value for each measured characteristic are italicized

Genes encoding endoglucanases belonging to families GH5, GH9, GH44 and GH128, xylanases to GH10, GH30, GH43 and GH115, polygalacturonases to GH28, β-mannanases to GH26 and GH53, endo-β-1,4-galactanases to GH51, α-glucuronidases to GH67, oligosaccharide degrading enzymes to GH35 and GH43, and starch degrading enzymes to GH13, GH27, GH77, GH88 and GH97 were all over-represented in the camel rumen’s metagenome (Additional file 2: Table S1). However, those encoding endoglucanases belonging to GH45 were under-represented in the camel rumen, but over-represented in the bovine rumen (Additional file 2: Table S1), while genes encoding α-*N*-acetylgalactosaminidases (GH109) were highly represented in the biogas metagenomes. Genes encoding oligosaccharide degrading enzymes belonging to GH2 and GH3, which are known to be involved in the degradation of microbial polysaccharides, were strongly represented in the rumen metagenomes but less so in the biogas metagenomes (Additional file 2: Table S1). In contrast, the biogas metagenomes featured an over-representation of genes encoding enzymes belonging to GH15, GH18, GH38, GH65, GH103 and GH116.

### CBMs

The CBM domain facilitates the binding of a CAZyme to its carbohydrate substrate, thereby influencing the enzyme’s catalytic activity. The set of predicted CBMs comprised 78 families. Those able to bind cellulose were encoded by 801 genes, including CBM6 (416 genes), CBM2 (295 genes), along with CBM1, CBM3, CBM8, CBM10, CBM11, CBM13, CBM17 and CBM28. Those binding xylan included CBM4 (462 genes) and CBM9 (275 genes), along with CBM15, CBM16 and CBM22; the chitin binding enzymes were distributed between CBM5, CBM12, CBM14, CBM18 and CBM19; with respect to the enzymes binding starch, the predominant CBM was CBM20 (307 genes), but genes encoding CBM21, CBM34 and CBM53 were also present; similarly, among the genes encoding proteins binding to galactose (CBM32 and CBM51), most encoded CBM32 domain enzymes. The camel rumen metagenome was notably enriched for genes encoding CBMs interacting with cellulose (CBM1, CBM2 and CBM13) (Additional file 2: Table S2). Among the xylan-binding domains, members of the CBM4 family were highly represented, which was not the case for the microbiome developed in either an anaerobic digester or a biogas fermenter. Also frequent in the camel rumen’s microbiome were CBM20, CBM37, CBM56, and CBM61 encoding genes. The most abundant single CBM was CBM50 (1732 genes), which was also common in the biogas fermenter and anaerobic digester microbiomes. Sequences encoding CBM54 xylan-binding domains and CBM25 starch-binding domains were notably over-represented in the biogas and anaerobic digester metagenomes.

### PULs

Clusters of co-localizing and co-regulated genes present specifically in *Bacteroidetes* species, termed PULs, encode multiple proteins involved in the detection, sequestration, hydrolysis and transport of complex carbohydrates [46, 47]. PULs were strongly represented in the camel rumen metagenome. The most abundant ones encoded the starch utilization system enzymes SusC (7016 sequences) and SusD (3530 sequences), along with the related enzymes SusD-like (215 sequences), SusD-like_2 (239 sequences) and SusD-like_3 (1947 sequences).

### CEs

The predicted CEs belonged to 15 families, of which the most frequently encountered were CE1 esterases (3628 sequences) and CE10 arylesterases (3211 sequences). Members of the families CE1, CE2, CE3, CE4, CE7, CE12 and CE13, which are known to play a critical role in hemicellulose degradation by enhancing xylan solubilization, were also relatively abundant. Along with the moose rumen and the elephant feces microbiome, that of the camel rumen was characterized by a particularly high representation of CEs (Additional file 2: Table S3).

### Cellulosome-associated domains (AAs, dockerins, and cohesins)

The auxiliary activity enzymes (AAs), which associate with lignin degradation were also strongly represented (Additional file 2: Table S4), and were categorized into seven families, of which AA6 (1,4-benzoquinone reductase) accounted for the product of > 90% of the 1319 sequences. This class of enzyme is also common in the moose metagenome and both the anaerobic digester and biogas fermenter microbiomes, but not in the bovine rumen’s. The presence of a large number of genes encoding either a cohesin or a dockerin domain is consistent with the enhanced capacity of the camel’s rumen microbiome to degrade lignocellulose mediated by cellulosomes (Additional file 2: Table S5). The representation of genes encoding dockerin contacting proteins was as high in the camel as in the moose rumen’s metagenome, but they were significantly less strongly represented (FDR-corrected *p* value < 0.001) in those associated with the bovine rumen, with elephant feces and in the biogas reactors. A total of 113 of the predicted GHs also harbored dockerin domains, suggesting their potential for interacting with cellulosomes. The GH families involved were GH13 and GH43 (each 13 genes), GH53 and GH124 (11 genes), GH128 (seven genes), GH5 and GH73 (six genes), GH16 and GH18 (five genes), GH11, GH26, GH30 and GH31 (four genes), and GH10, GH32 and GH95 (genes). In addition, 178 CBMs, 23 CEs, 14 PLs and one AA protein also harbored dockerin domains.

The presence of 1092 sequences harboring an SLH domain provided additional evidence for active cellulosome-mediated plant cell-wall degradation in the camel rumen. The analysis also identified 675 putative scaffoldin proteins, of which 55 were homologs of *C. cellulovorans* CbpA, 304 of *B. cellulosolvens* ScaB, 28 of *C. cellulolyticum* CipC, four of *R. flavefaciens* ScaB, five of *C. acetobutylicum* CipA and 204 of *P. cellulosolvens* ScaA.

### Taxonomic origin of the predicted CAZymes

**Fig. 1:**
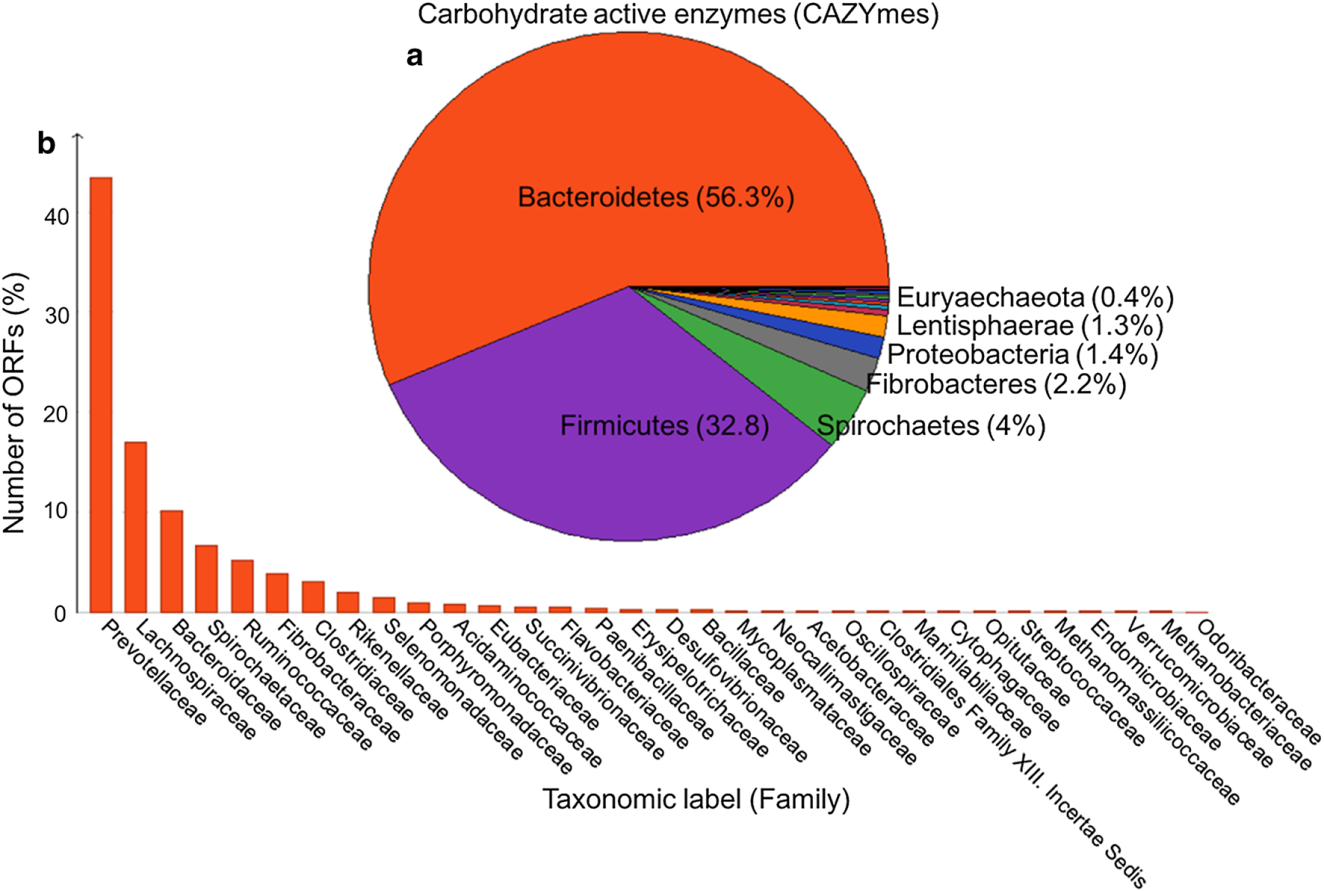
Phylum (**a**) and family (**b**) level taxonomic distribution of the predicted carbohydrate active enzymes (CAZymes) including GHs, GTs, CBMs, CEs, PLs, AAs, dockerins, and cohesins. The predicted ORFs were blast searched against the most recent version of non-redundant protein (NR) database with an *e*-value cutoff 1e−3 and num_alignments 20. Taxonomic affiliates were inferred using the lowest common ancestor (LCA) algorithm of MEGAN (version 6.7.19) [38]

**Fig. 2:**
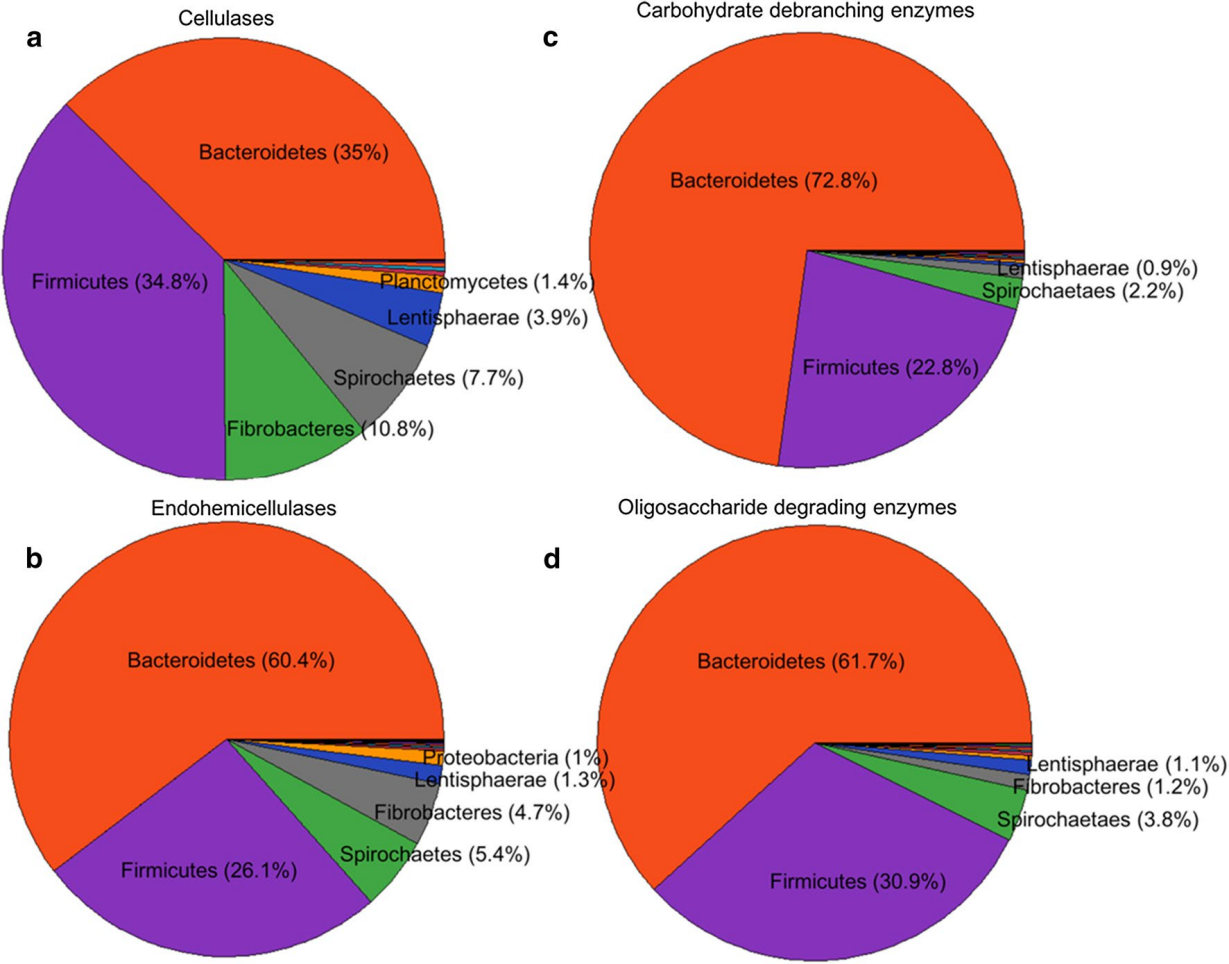
Phylum level taxonomic origin of the predicted cellulases belonging to families GH5, GH9, GH44, GH45, GH48, and GH74 (**a**), endohemicellulases from families GH8, GH10, GH11, GH12, GH26, GH28, and GH53 (**b**), carbohydrate debranching enzymes from families GH51, GH54, GH67, and GH78 (**c**), and oligosaccharide degrading enzymes including families GH2, GH3, GH29, GH35, GH38, GH39, GH42, GH43, and GH94 (**d**). The predicted ORFs were blasted against the most recent version of the NR database with an *e*-value cutoff 1e−3 and num_alignments 20. Taxonomic affiliates were assigned according to the lowest common ancestor (LCA) algorithm of MEGAN

Inference of the taxonomic origin of the rumen’s microbiome components based on the gene sequences encoding CAZymes implied the presence of 19 phyla of microbes. Species belonging to the *Bacteroidetes* (56.3%), the *Firmicutes* (32.8%), the *Spirochaetes* (4.0%), the *Fibrobacteres* (2.2%), the *Proteobacteria* (1.4%), the *Lentisphaerae* (1.3%), the *Euryarchaeota* (0.4%) and the *Verrucomicrobia* (0.3%) collectively represented 98.7% of sequences (Fig. 1). The distribution of these abundant species at the family level is given in Fig. 1b. Based on the 16S rRNA sequence data, species belonging to the *Bacteroidetes* and *Firmicutes* phyla accounted for 60% of rumen’s microbiome, but on the basis of CAZymes, their joint contribution was estimated to be > 89%. An inspection of the cellulase sequences (families GH5, GH9, GH44, GH45, GH48 and GH74) suggested an almost equal contribution of the *Bacteroidetes* and *Firmicutes* phyla in rumen’s metagenome cellulase repertoire (35 and 34.8%, respectively), despite the observation that their relative abundance was significantly different (Fig. 2a). Over 10% of the cellulases were encoded by *Fibrobacteres* species whose abundance was estimated to be low. *Spirochaetes* and *Lentisphaerae* species were responsible for, respectively, 7.2 and 3.6% of the cellulase-encoding genes. A similar analysis focusing on the endohemicellulases (families GH8, GH10, GH11, GH12, GH26, GH28 and GH53), as illustrated in Fig. 2b, revealed that > 60% of this class of enzyme was produced by *Bacteroidetes* species, although *Firmicutes* species also made a substantial contribution (> 26%), with smaller contributions from *Spirochaetes* (5.4%) and *Fibrobacteres* (4.7%) species. The source of the genes encoding carbohydrate debranching enzymes (families GH51, GH54, GH67 and GH78) was largely (72.7%) from *Bacteroidetes* species, with a substantial contribution (22.7%) from *Firmicutes* species (Fig. 2c). A similar distribution was observed with respect to the genes encoding oligosaccharide-degrading enzymes (families GH2, GH3, GH29, GH35, GH38, GH39, GH42, GH43 and GH94): 61.8% of the sequences were contributed by *Bacteroidetes* species and 30.9% by *Firmicutes* species, with a minor contribution from species belonging to either the *Spirochaetes* or the *Fibrobacteres* (Fig. 2d). With respect to the sequences encoding AA domains, > 48% were contributed by *Firmicutes* species and 35% by *Bacteroidetes* species (Additional file 1: Figure S1). The proportion of the set of AA sequences encoding vanillyl alcohol oxidase, VAOs, (AA4) and 1,4-benzoquinone reductase (AA6) was 6.4%, and these originated from species belonging to the *Euryarchaeota,* and therefore, may play a key role in methane metabolism. Interestingly, VAOs are biotechnologically relevant enzymes capable of catalyzing oxidation, deamination, demethylation, dehydrogenation, and hydroxylation reactions on a wide range of phenolic compounds [48].

### The potential hydrolytic capacity of the camel rumen’s microbiome

**Fig. 3:**
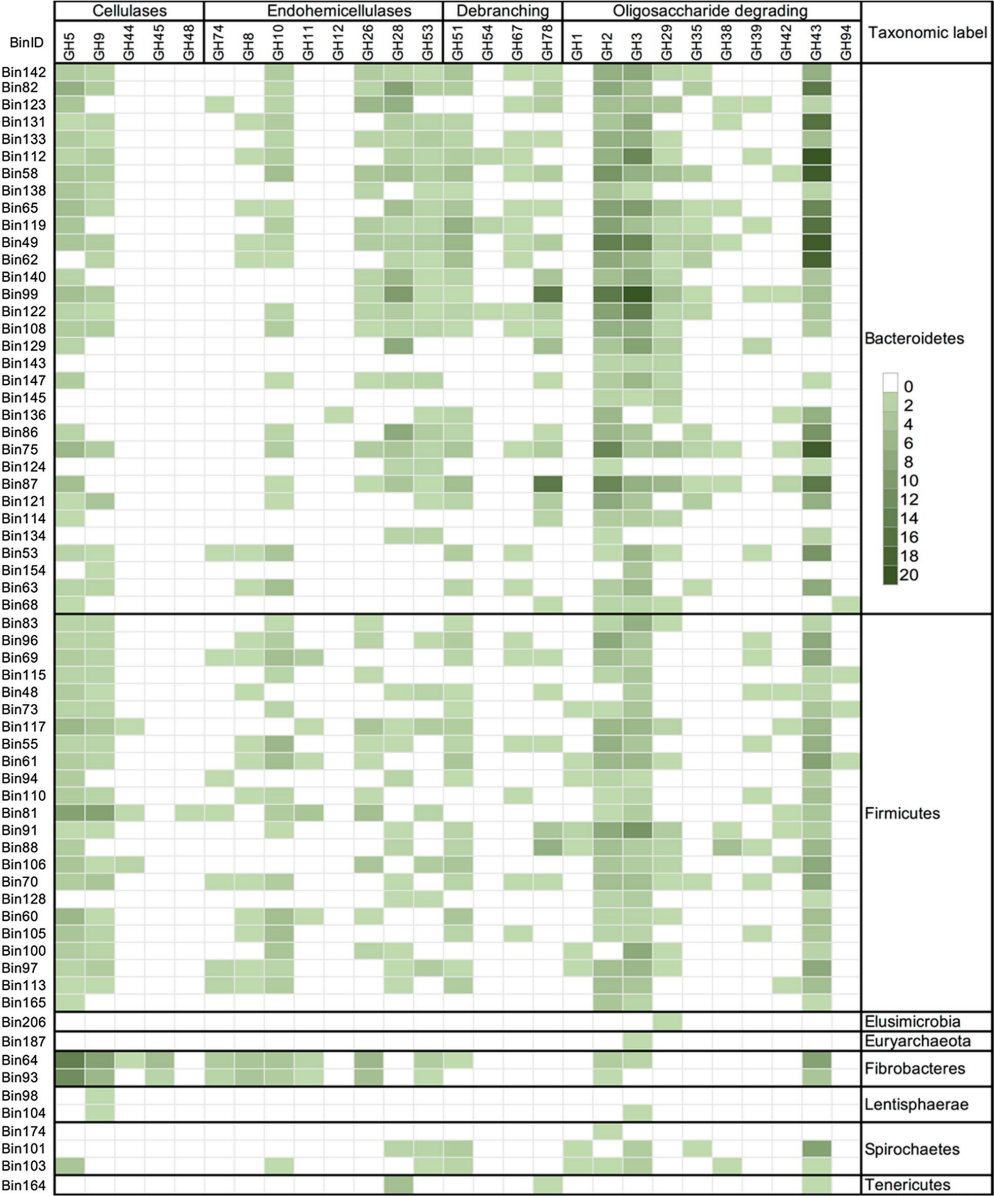
Heatmap shows the distribution of major lignocellulose degrading enzymes in the reconstituted genome bins. GHs were grouped according to their substrate utilization and carbohydrate degrading activities on major components of plant cell walls. The phylum-level classification of the GBs is shown on the right-hand side of the panel. *Bacteroidetes* and *Firmicutes* bins shows the greatest diversity and abundance for GHs

The binning process enabled inferences to be drawn regarding which microbial species were responsible for lignocellulose hydrolysis and fermentation. Species belonging to the two predominant phyla *Bacteroidetes* and *Firmicutes* contributed, respectively, 84 and 56 bins which featured lignocellulose degrading enzymes. In the *Bacteroidetes* bins, 44% of all predicted GH genes encoding products within the GH2, GH3, GH13, GH28, GH43, GH92, GH97 and GH109 families, while among the *Firmicutes* bins, > 47% of the sequences encoded products belonging to GH2, GH3, GH5, GH10, GH13, GH43 and GH109. The *Bacteroidetes* enzymes involved mainly oligosaccharide or hemicellulose degraders (GH2, GH3 and GH43) and hemicellulases (GH10, GH28 and GH53) (Fig. 3), but polysaccharide debranching enzymes belonging to GH51, GH54, GH67 and GH78 were also relatively abundant. Cellulases were less well represented, with only members of GH5 and GH9 being detected. Based on their substrate utilization, the *Firmicutes* GHs were in the main concerned with either oligosaccharide (families GH1, GH2, GH3, GH29, GH39 and GH43) or cellulose (GH5, GH9, GH44 and GH74) degradation. The *Firmicutes* species harbored numerous genes encoding hemicellulases (GH8, GH10, GH11, GH26, GH28 and GH53). The implication is that the *Bacteroidetes* and *Firmicutes* species act synergistically to degrade lignocellulosic material.

The two *Fibrobacteres* bins #64 and #93 which were identified as *F. succinogenes* harbored an abundance of genes encoding either cellulases or hemicellulases but lacked the most GHs encoding oligosaccharide degrading and debranching enzymes. Their cellulases belonged to families GH5, GH9, GH45 and GH74, while their hemicellulases belonged to families GH8, GH10, GH11, GH26 and GH53. They also lacked GH48 and GH12 endoglucanases and GH28 polygalacturonases, along with the cohesin and dockerin domains characteristic of cellulosomal structures. However, they were diverse with respect to both CBMs and PLs. *F. succinogenes* is a fibrolytic and pectin-degrading species capable of utilizing cellulose as its sole energy source [49, 50], highlighting its potential as a lignocellulose degrader in the camel rumen. The *Spirochaetes* bins (#174, #101 and #103) harbored genes encoding members of 36 GH families, of which the most frequently occurring were GH3, GH13, GH23, GH43, GH51, GH57 and GH77. The bins assigned to *Elusimicrobia* (#206), *Tenericutes* (#164) and *Lentisphaerae* (#98 and #104) showed only a limited degree of diversity with respect to the GHs represented; they lacked the GHs contributing most strongly to lignocellulose degradation, with only a single GH9 member detected in each of the two *Lentisphaerae* bins. This finding is in accord with previous study on an anaerobic digester that showed the limited diversity of GHs in *Lentisphaerae* GBs, suggesting that they likely contribute little to lignocellulose hydrolysis [23]. The *Elusimicrobia* and *Euryarchaeota* bins harbored the fewest GHs (four and two, respectively), suggesting that these species contributed little to lignocellulose degradation. The single bin affiliated to *Tenericutes* contained sequences predicted to encode four CBMs, nine PLs and ten GHs (mostly members of GH28). A search for cohesin sequences identified the domain in certain species of *Bacteroidetes* and *Firmicutes*. A characteristic feature of scaffoldin proteins, a major component of cellulosomal assemblies, is the presence of multiple repeated cohesin domains, a structure which serves as a docking site for the attachment of catalytic CAZymes [51]. Several of the bins (#60, #73, #81, #150, #106 and #136) included genes encoding cohesins comprising between two and four tandemly arranged cohesin domains. Bin #81, assigned to the genus *Ruminococcus*, harbored four cohesin encoding sequences, three of which included two cohesin domains and one three tandemly repeated cohesin domains. Additionally, there were 21 genes encoding dockerin domains, confirming the likely involvement of the products of this bin’s genes in cellulosome-mediated plant cell-wall degradation. There was also an array of GH-encoding genes present (67 candidates), most of which encoded cellulases belonging to the families GH5 (nine genes), GH9 (nine genes) and GH43 (four genes), along with hemicellulases belonging to families GH3, GH10 and GH13 (three genes in each case). Among the CAZymes produced by members of this bin were a single member of each of the families GH5, GH26, GH30 and GH127 and two of family GH43; their harboring of a PL1 dockerin domain suggested them as likely components of the cellulosome. This was the only bin which contained a single gene encoding a GH48 family cellulase, a potent enzyme capable of degrading crystalline cellulose; a similar result has recently reported for the moose rumen metagenome [39]. *Ruminococcus* species are the only known rumen microbes which carry only a single gene encoding a family GH48 enzyme [52, 53]. A recent transcriptome analysis of the bovine rumen microbial community has shown that transcripts from family GH48-encoding genes are amongst the most abundant of all transcripts present in animals fed a fiber-rich diet [54]. The *Ruminococcus* species *R. albus* and *R. flavefaciens* are both prominent lignocellulose degraders present in the rumen [55]. A complete set of genes required for the assembly of cellulosomal-like structures has recently been reported for *R. flavefaciens* [56], a species which harbors genes encoding a diversity of dockerin- and cohesin-containing proteins [55]. Sequences specifying dockerin domains were abundant in the *Bacteroidetes* bins #49, #58 and #136 (Table 1). There were also several cases in which a single protein contained multiple dockerin domains, consistent with their function as adaptor proteins. The presence of dockerin domains in contigs from *Bacteroidetes* has been recently reported in both the bovine and the moose rumen metagenomes [39, 57], implying that the ability to degrade cellulosome-based lignocellulose is a general feature of the rumen microbiome. However, recent evidence has also turned up dockerin domains in enzymes not known to be associated with fiber degradation [57].

### Identification of PULs in the genome bins

**Fig. 4:**
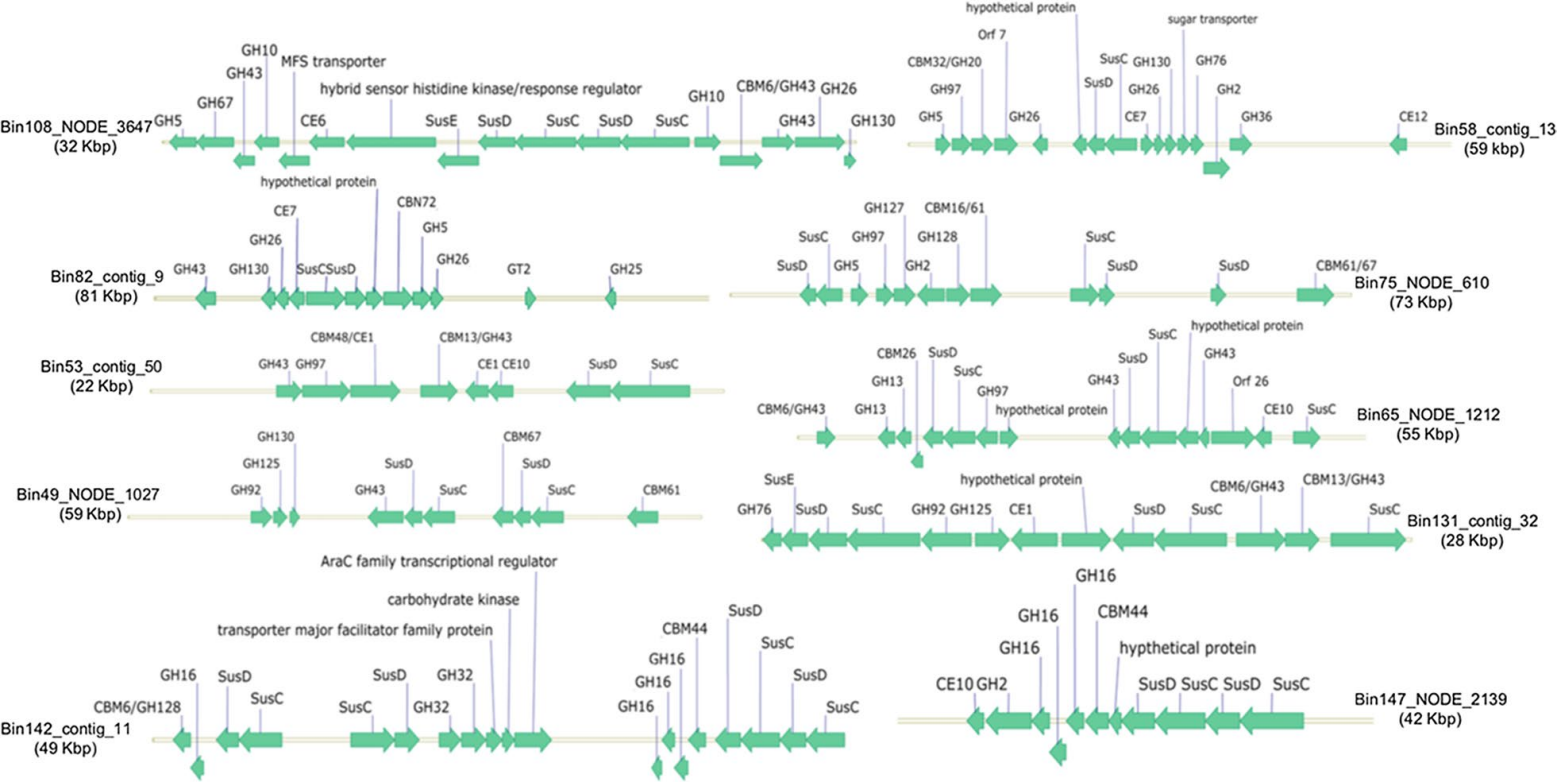
The organization of genes encoding CAZymes within PULs. In addition to those encoding CAZymes, genes encoding transporters, receptors (such as a hybrid sensor histidine kinase/response regulator), gene regulatory proteins (such as an AraC transcription regulator) and carbohydrate kinases are also present. Some PULs also contained genes encoding for proteins of unknown function suggesting that they are likely CAZymes which have been remained to be characterized. Additional examples are presented in Additional file 1: Figure S2

A PUL refers to a set of physically-linked and functionally-related genes organized around a SusC-SusD gene pair that facilitate the utilization of a particular polysaccharide substrate [47]. Potential PULs were identified by searching within contigs for tandem pairs of SusC-SusD, then extending the search to identify nearby genes encoding other CAZymes. The scan revealed 409 PULs, distributed over 28 of the 32 *Bacteroidetes* bins, showing them to be a common feature of the camel rumen *Bacteroidetes* community. The four *Bacteroidetes* bins which lacked any PUL representation were #68, #114, #143 and #145. The number of PULs per bin varied from 2 to 35 (mean 14). Those harboring the highest number of PULs were the two genus *Prevotella* bins #65 (35 PULs) and #49 (26 PULs), along with bins #123 (26 PULs) and #108 (25 PULs), suggesting their high polysaccharide degrading capacity. The gene organization around a SusC-SusD gene pair has been depicted for a sample of bins in Fig. 4 and Additional file 1: Figure S2.

The most common carbohydrate degrading enzymes associated with the PULs were α and β-glucosidases, β-galactosidases (GH35), endoglucanases (GH5, GH9), polygalacturonases (GH28), β-mannosidases (GH2), endo-β-1,4-xylanases (GH30), rhamnosidases (GH106), and β- mannanases (GH26) implying that they likely have a broad substrate specificity. The presence of genes encoding carboxyl esterase (CE10), pectin acetylesterase (CE12), pectate lyase (PL1) and rhamnogalacturonan endolyase (PL11) was also noted among the PULs, suggesting that they have been tailored for the breakdown of complex lignocellulosic polysaccharides. An illustrative example of these clusters, including genes encoding enzymes able to perceive, degrade and transport a hemicellulosic polysaccharide, is depicted in Fig. 4. A similar cluster, composed of susC-susD-susC-susD-susE and genes encoding CE6, and members of families GH10, GH43, GH67 and GH5, has been reported as present in the genome of the cellulolytic species *Bacteroides cellulosilyticus* [47]. Other examples include bin #129, comprising a cluster of genes encoding a number of pectin-targeting enzymes (PL1, GH105, CE8 and GH28) (Additional file 1: Figure S2), and bins #142 and #147, where genes encoding multiple members of GH16 were linked to those encoding CBM44 and a GH2 family member for targeting xyloglucan substrates (Fig. 4).

### The contribution of the camel rumen’s microbiota to VFA production

Since the main VFAs produced by ruminants are acetate, propionate and butyrate, the presence of key marker genes for the synthesis of these compounds was investigated in the reconstituted genome bins. Genes encoding enzymes involved in acetate production were represented in most of the bins, as shown by the presence of *ackA* (encoding acetate kinase) and *pta* (phosphoacetyltransferase) in fully 92% of the bins. Only two of the *Bacteroidetes* bins (#75 and #108), the *Elusimicrobia* bin #206 and the *Euryarchaeota* bin #187 lacked a representation of either *ackA* and/or *pta*. Of the three pathways involved in propionate production [58], the only one detected as present in the rumen was the succinate pathway: the diagnostic gene for this pathway encodes methylmalonyl CoA decarboxylase. Evidence for propionate production via either the acrylate or the propanediol pathways was lacking, because of the failure to detect any of the marker genes *lcdA* (acrylate pathway), *pduP* or *pduQ* (propanediol pathway). Similarly, neither of these two pathways develop in an anaerobic digester [23], while in the human gut microbiome, propionate production achieved via propanediol and acrylate pathways has been reported, although the dominant pathway is thought to be via succinate [58]. *Bacteroidetes* species made the largest contribution to propionate production: out of the 32 *Bacteroidetes* bins, only four (#82, #121, #136 and #145) lacked an *mmdA* gene. For these latter four bins, the failure to detect *mmdA* may have been artefactual, given that the genome sequences were less than 100% complete. Propionate production was a common feature of the *Firmicutes* bacteria, but not throughout the phylum, since only 14 of the 23 *Firmicutes* bins harbored *mmdA* sequences. The marker gene was also detected in the bins allocated to either *Spirochaetes* or *Fibrobacteres*, while it was absent from bins assigned to either *Tenericutes*, *Lentisphaerae*, *Elusimicrobia* or *Euryarchaeota*.

Butyrate production is believed to derive from two distinct pathways [59]. These two routes are distinguished from one another during the formation of butyrate from butyryl-CoA during which alternative enzymes butyrate kinase (*buk*) or butyrylCoA:acetate CoA-transferase (*but*) act by utilizing different substrates. A search for the occurrence of *buk* and *but* homologs revealed the presence of multiple presumptive butyrate producers belonging to the phyla *Bacteroidetes*, *Firmicutes* and *Lentisphaerae*. Nine of the *Bacteroidetes* bins harbored *buk* sequences, and 16 harbored *but* sequences, suggesting a predominance for the latter pathway. In the *Firmicutes* species, however, butyrate production was dominated by the *buk* pathway, as 14 bins contained *buk* sequences, while only four contained *but* sequences. The *Lentisphaerae* bins harbored exclusively *but* sequences.

## Discussion

This paper has presented a first analysis of the sequencing-based characterization of the camel rumen’s metagenome. Inferring the identity of its microbial components from metagenomic sequences has shown that the microbiome is dominated by taxa belonging to the phyla *Bacteroidetes* and *Firmicutes*, with some minor representation of taxa belonging to the phyla *Verrucomicrobia*, *Spirochaetes*, *Proteobacteria*, *Fibrobacteres*, *Tenericutes* and *Lentisphaerae*. On this basis, the conclusion is that from a taxonomical viewpoint, the species composition of the camel rumen is not so dissimilar to that of either the bovine [13, 16, 20] or the moose [39, 60] rumen. A notable difference, however, was the relatively strong representation of *Fibrobacteres* species, known to target plant fiber and pectin [49, 50]. The presence of these microbes may reflect the uniqueness of the camel’s diet, which typically includes material having a high content of lignocellulose. Similarly, the camel rumen hosted a number of *Spirochaetes* species, which are also to be found in other lignocellulose degrading environments, such as the moose rumen [39] and the termite hindgut [61]. This data along with the relatively high proportion of GHs assigned to members of this phylum (as discussed below) suggests that they likely have a significant contribution to lignocellulose degradation in the camel rumen. The COG classification of the metagenome likewise demonstrated a high representation (> 9%) of genes encoding proteins involved in carbohydrate transport and metabolism, commensurate with the rumen fermentation process.

Compared to the bovine and moose rumen [16], elephant feces [15], and biogas fermenters [21, 22] metagenomes, the camel rumen’s microbiome harbored a higher number of genes encoding GHs (Table 2). With respect to its CAZyme profile, the camel rumen was very similar to that of the moose rumen, but was significantly different from those of the bovine rumen, elephant feces and biogas reactors (Additional file 2: Tables S1–S6). This difference likely reflects the high lignocellulose content of the camel (and moose) diet [39, 60], which is arguably the major driver of the rumen’s microbial composition [29]. The camel rumen’s metagenome also harbored an array of sequence features unique to the cellulosome complex, an elaborate multi-enzyme assembly that allows efficient decomposition of plant lignocellulosic materials. The cellulosome complex consists of a non-catalytic scaffoldin protein which includes a cellulose-binding domain(s) or CBMs to enable substrate binding, along with a cohesin domain(s) to facilitate its binding to various catalytic CAZymes via their dockerin domains [62]. Anaerobic species belonging to both *Firmicutes* and *Bacteroidetes* are known to utilize this system to degrade plant cell-wall polysaccharides [62, 63]. The picture which emerged was that the camel rumen’s microbiome has adapted itself for the degradation of plant lignocellulosic biomass, driven by the fact that this type of material representing the bulk of the camel diet.

The predicted GHs were encoded by genes harbored by species belonging to the phyla *Bacteroidetes* (61%), *Firmicutes* (29%), *Spirochaetes* (4%), *Fibrobacteres* (2%), *Lentisphaerae* (1%), *Proteobacteria* (1%) and *Verrucomicrobia* (< 1%) (Additional file 1: Figure S1), so the inference is that > 90% of the lignocellulose degradation potential is fueled by species belonging to just two phyla, which also accounted for > 80% of the microbes found to adhere to fibers in the rumen [10]. While it is well recognized that species belonging to the phyla *Bacteroidetes*, *Firmicutes* and *Fibrobacteres* are the major agents of lignocellulose degradation in the rumen [64, 65], species belonging to both the *Spirochaetes* and *Lentisphaerae* phyla also contributed a minor proportion of the GHs. A similar analysis focused on cellulases showed that *Bacteroidetes* and *Firmicutes* species made an equal contribution (each 37%), with the remainder of the genes originating from species belonging to either the *Fibrobacteres* (10%) or the *Spirochaetes* (4%) phyla, which are known to have a significant contribution to lignocellulose degradation [66]. The pronounced representation of *Fibrobacteres* species and their particular contribution to the CAZyme profile (specifically with respect to cellulase) (Fig. 2) highlights their key involvement in lignocellulose degradation in the camel rumen. Compared to the bovine rumen’s microbiome [19], the contribution of *Bacteroidetes* species to the production of CAZymes in the camel rumen was relatively high (56% vs 40%) (Fig. 1), but in contrast, while in the bovine rumen, *Proteobacteria* accounted for > 10% of GH-encoding sequences, the equivalent frequency in the camel rumen was just 1.4%. Similarly, in biogas fermenters and other engineered environments, the contribution of *Proteobacteria* to carbohydrate degrading enzymes is high, while *Bacteroidetes* species make only a limited contribution [21–23, 67]. This data points on the unique carbohydrate degrading capability of the camel’s rumen microbiome, as members of the phyla *Bacteroidetes* and *Firmicutes* are known as potent lignocellulose degraders which their association with lignocellulose degradation has been well established in the bovine rumen [13].

In the light of methodological advances in metagenome assembly and binning, it has now become possible to assemble draft genomes for most members of the rumen microbiome, even for those species which represent only a small proportion of the overall community, as well as for those which are currently not represented in databases, even by related species [20]. This capacity should facilitate a better understanding of both the diversity and the function of the rumen microbiome than has been possible using a conventional culture-based approach. The combined composition and coverage-based binning of the DNA scaffolds deduced from the metagenomic sequence defined 65 bins, about two-thirds of which were associated with species belonging to either the *Bacteroidetes* or the *Firmicutes* phyla (Table 1). In addition to a high level of functional redundancy with respect to their lignocellulose degrading capability and VFA fermentation ability, the camel rumen’s microbiome also displayed a degree of diversity with respect to types of lignocellulosic substrate. Members of the *Bacteroidetes* phylum showed a broad substrate specificity as they contained a varied combination of CAZymes in the form of PULs, which are clusters of genes encoding catalytic CAZymes, sugar transporters and regulatory proteins, required for the sequestration, break-down and transport of glycan substrates [46, 47]. The present data suggest that the camel rumen exploits *Bacteroidetes* PUL enzymes to assimilate complex dietary carbohydrates.

The decomposition by rumen microbes of lignocellulosic material and the fermentation of the sugars released thereby to form short chain VFAs provides a major benefit to the ruminant host. Therefore, the capability of the rumen’s microbiome to convert plant lignocellulosic material into VFAs is very important for the nutrition of the host ruminant, because these small metabolites are readily absorbed into the host’s bloodstream and assimilated as main source of nutrient [7, 68]. Up to 80% of the host’s energy requirement is supplied by VFAs produced in either the rumen or the large intestine [7]. VFAs also serve as building blocks for the production of milk, and are essential for the normal function of the intestinal epithelium [68]. Screening for VFA fermentation pathways showed that acetate production was a common feature of nearly all the components of the rumen microbiome (the exceptions relate to members of the phyla *Elusimicrobia* and *Euryarchaeota*). Propionate production is carried out largely via succinate, since there is at best only fragmentary evidence for its production via either the acrylate or the propanediol pathways. In the human gut, microbial species belonging to the phylum *Bacteroidetes* are responsible for much of the acetate and propionate production which occurs, while butyrate production is handled by *Firmicutes* species [69, 70]. In the camel rumen, however, *Bacteroidetes* and *Firmicutes* species have an equal capability for acetate and propionate production. Likewise, butyrate production was found to occur in 24 out of 35 *Bacteroidetes* (totaling 75%) and 16 out of 24 *Firmicutes* bins (66%) indicating that both are equally able to produce butyrate but likely through alternative pathways.

Overall, it was clear that the metagenomics approach represents an effective means of analyzing the functional potential of the rumen’s microbiome, which is so important for both the health and productivity of ruminant animals. Combining metagenomic data with metatranscriptomic, metaproteomic and metabolomic data should shed even more light on the various contributions of the rumen’s microbiome. The GHs present in the camel rumen shared an average sequence identity of 70% with their homologs, consistent with a recent analysis of bovine rumen CAZymes [20], which suggested that a number of the enzymes may exhibit interesting biochemical properties. For example, our group have recently cloned and enzymatically characterized a novel cold-adapted endoglucanase (CelCM3) from the camel rumen dataset [71]. Interestingly, it showed a 50% activity at 4 °C and displayed tolerance to metal ions, non-ionic detergents, urea and organic solvents, suggesting its potential to be used in processes that need to run at moderately low temperatures. These data have highlighted that the camel’s rumen microbiome harbors an as yet largely untapped source of enzymes with an unknown potential which could be exploited to improve a range of biotechnological processes including biofuel and food processing industries.

## Additional files

**Additional file 1: Figure S1.** The taxonomic distribution of GHs (A), PLs (B), CBMs (C), AAs (D), and CEs (E) predicted in the camel rumen’s metagenome. **Figure S2.** Additional examples of PULs identified in the *Bacteroidetes* bins reconstituted from the camel rumen’s metagenome.

**Additional file 2: Table S1.** Details of the number of predicted glycoside hydrolases (GHs) detected in the assembled metagenomes (contigs ≥ 1000 bp). The abundances of GHs were compared between variously sourced metagenomes including the bovine and the moose rumen, elephant faces, and the biogas reactors. Statistical significant differences in CAZyme profiles between the camel rumen’s metagenome and the other metagenomes were assessed using Fisher’s exact test. *P* values were corrected using FDR method. ns = non-significant, * = FDR-corrected *p* value < 0.05, ** = FDR-corrected *p* value < 0.01, *** = FDR-corrected *p* value < 0.001. **Table S2.** Counts of carbohydrate binding modules (CBMs) containing proteins predicted in the assembled metagenomes (contigs ≥ 1000 bp). Statistical significant differences in CAZyme profiles between the camel rumen’s metagenome and the other metagenomes were assessed using Fisher’s exact test. *P* values were corrected using FDR method. ns = non-significant, * = FDR-corrected *p* value < 0.05, ** = FDR-corrected *p* value < 0.01, *** = FDR-corrected *p* value < 0.001. **Table S3.** The predicted carbohydrate esterases (CEs) characterized in the assembled metagenomes (contigs ≥ 1000 bp). Statistical significant differences in CAZyme profiles between the camel rumen’s metagenome and the other metagenomes were assessed using Fisher’s exact test. *P* values were corrected using FDR method. ns = non-significant, * = FDR-corrected *p* value < 0.05, ** = FDR-corrected *p* value < 0.01, *** = FDR-corrected *p* value < 0.001. **Table S4.** Table shows the auxiliary activity domain containing proteins (AAs) identified in the assembled metagenomes (contigs ≥ 1000 bp). Statistical significant differences in CAZyme profiles between the camel rumen’s metagenome and the other metagenomes were assessed using Fisher’s exact test. *P* values were corrected using FDR method. ns = non-significant, * = FDR-corrected *p* value < 0.05, ** = FDR-corrected *p* value < 0.01, *** = FDR-corrected *p* value < 0.001. **Table S5.** Table presents the distribution of dockerin, cohesion, and surface layer homology (SLH) domain containing proteins predicted in the assembled metagenomes (contigs ≥ 1000 bp). Statistical significant differences in CAZyme profiles between the camel rumen’s metagenome and the other metagenomes were assessed using Fisher’s exact test. *P* values were corrected using FDR method. ns = non-significant, * = FDR-corrected *p* value < 0.05, ** = FDR-corrected *p* value < 0.01, *** = FDR-corrected *p* value < 0.001. **Table S6.** The numbers of predicted polysaccharide lyases (PLs) detected in the assembled metagenomes (contigs ≥ 1000 bp). Statistical significant differences in CAZyme profiles between the camel rumen’s metagenome and the other metagenomes were assessed using Fisher’s exact test. *P* values were corrected using FDR method. ns = non-significant, * = FDR-corrected *p* value < 0.05, ** = FDR-corrected *p* value < 0.01, *** = FDR-corrected *p* value < 0.001.
