# Supplementary material for: A metagenomic analysis of the camel rumen’s microbiome identifies the major microbes responsible for lignocellulose degradation and fermentation
Source: Biotechnol Biofuels. 2018 Aug 2;11:216. doi: 10.1186/s13068-018-1214-9 (PMC6071333; doi:10.1186/s13068-018-1214-9)
Supplement: Supplementary file 1 — Additional file 1: Figure S1. The taxonomic distribution of GHs (A), PLs (B), CBMs (C), AAs (D), and CEs (E) predicted in the camel rumen’s metagenome. Figure S2. Additional examples of PULs identified in the Bacteroidetes bins reconstituted from the camel rumen’s metagenome. [file 13068_2018_1214_MOESM1_ESM.docx]

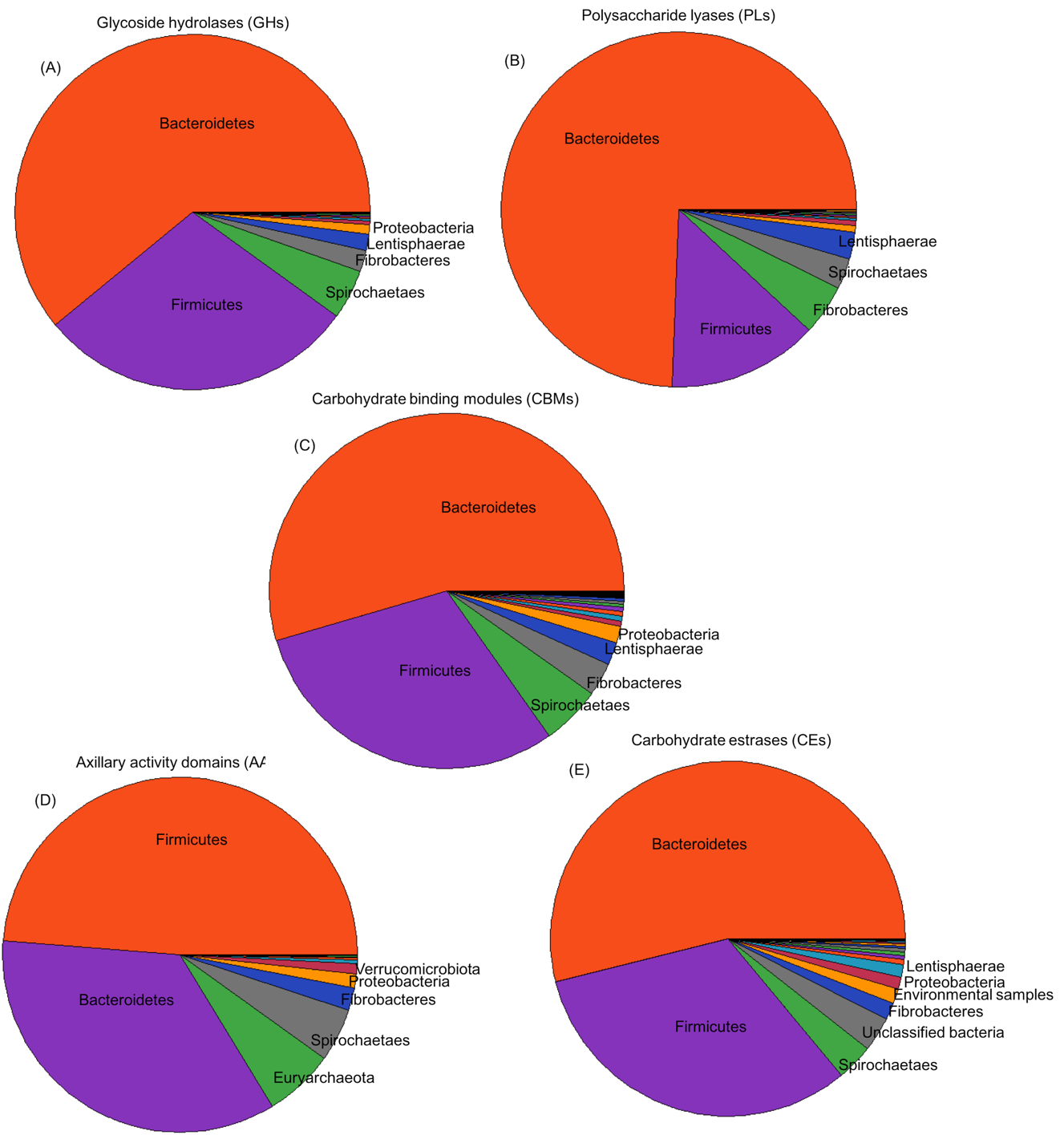


Figure S1: The taxonomic distribution of GHs (A), PLs (B), CBMs (C), AAs (D), and CEs (E) predicted in the camel rumen’s metagenome


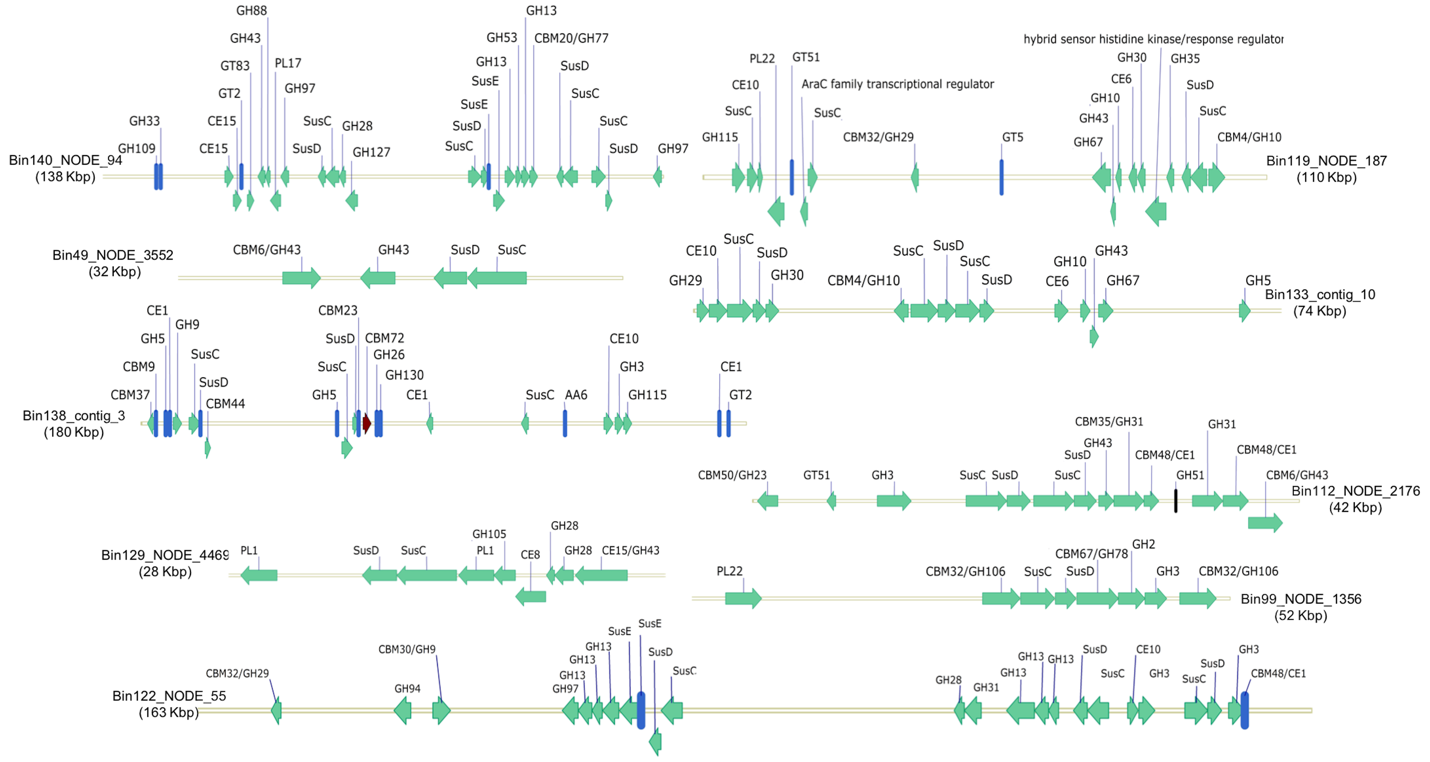


Figure S2: Additional examples of PULs identified in the *Bacteroidetes* bins reconstituted from the camel rumen’s metagenome.
