# Supplementary material for: A metagenomic analysis of the camel rumen’s microbiome identifies the major microbes responsible for lignocellulose degradation and fermentation
Source: Biotechnol Biofuels. 2018 Aug 2;11:216. doi: 10.1186/s13068-018-1214-9 (PMC6071333; doi:10.1186/s13068-018-1214-9)
Supplement: Supplementary file 2 — Additional file 2: Table S1. Details of the number of predicted glycoside hydrolases (GHs) detected in the assembled metagenomes (contigs ≥ 1000 bp). The abundances of GHs were compared between variously sourced metagenomes including the bovine and the moose rumen, elephant faces, and the biogas reactors. Statistical significant differences in CAZyme profiles between the camel rumen’s metagenome and the other metagenomes were assessed using Fisher’s exact test. P values were corrected using FDR method. ns = non-significant, * = FDR-corrected p value < 0.05, ** = FDR-corrected p value < 0.01, *** = FDR-corrected p value < 0.001. Table S2. Counts of carbohydrate binding modules (CBMs) containing proteins predicted in the assembled metagenomes (contigs ≥ 1000 bp). Statistical significant differences in CAZyme profiles between the camel rumen’s metagenome and the other metagenomes were assessed using Fisher’s exact test. P values were corrected using FDR method. ns = non-significant, * = FDR-corrected p value < 0.05, ** = FDR-corrected p value < 0.01, *** = FDR-corrected p value < 0.001. Table S3. The predicted carbohydrate esterases (CEs) characterized in the assembled metagenomes (contigs ≥ 1000 bp). Statistical significant differences in CAZyme profiles between the camel rumen’s metagenome and the other metagenomes were assessed using Fisher’s exact test. P values were corrected using FDR method. ns = non-significant, * = FDR-corrected p value < 0.05, ** = FDR-corrected p value < 0.01, *** = FDR-corrected p value < 0.001. Table S4. Table shows the auxiliary activity domain containing proteins (AAs) identified in the assembled metagenomes (contigs ≥ 1000 bp). Statistical significant differences in CAZyme profiles between the camel rumen’s metagenome and the other metagenomes were assessed using Fisher’s exact test. P values were corrected using FDR method. ns = non-significant, * = FDR-corrected p value < 0.05, ** = FDR-corrected p value < 0.01, *** = FDR-corrected p [file 13068_2018_1214_MOESM2_ESM.doc]

Table S1: the number of predicted glycoside hydrolases (GHs) detected in the assembled metagenomes (contigs ≥1000 bp). The abundances of GHs are compared between variously sourced metagenomes including camel rumen, bovine rumen, elephant faces, moose rumen, and biogas reactors. Statistical significant differences in CAZyme profiles between the camel rumen’s metagenome and the other metagenomes were assessed using Fisher’s exact test. P-values were corrected using FDR method. ns = non-significant, * = FDR-corrected p-value  0.05, ** = FDR-corrected p-value  0.01, *** = FDR-corrected p-value  0.001

| CAZyme family | Camel rumen (this study) | Cow rumen (Hess et al 2011) | Elephant feces (Ilberger et al 2014) | Biogas reactor (Campanaro et at 2016) | Biogas reactor (Gullert et al 2016) | Moose rumen  (Svartström et al 2017) |
| --- | --- | --- | --- | --- | --- | --- |
| GH1 | 103 | 87^ns^ | 93^ns^ | 95^ns^ | 119^ns^ | 22^ns^ |
| GH2 | 2273 | 885^***^ | 1454^**^ | 353^***^ | 696^***^ | 469^ns^ |
| GH3 | 2115 | 1552^***^ | 1109^***^ | 348^***^ | 692^***^ | 320^ns^ |
| GH4 | 211 | 192^ns^ | 200^ns^ | 190^ns^ | 281^***^ | 67^ns^ |
| GH5 | 1382 | 866^***^ | 871^*^ | 180^***^ | 400^***^ | 206^ns^ |
| GH6 | 0 | 0 | 0 | 0 | 1^ns^ | 0 |
| GH8 | 172 | 196^*^ | 103^ns^ | 29^**^ | 59^**^ | 18^ns^ |
| GH9 | 612 | 396^***^ | 244^***^ | 86^***^ | 168^***^ | 65^*^ |
| GH10 | 689 | 473^***^ | 376^**^ | 122^***^ | 226^***^ | 104^ns^ |
| GH11 | 63 | 116^***^ | 27^ns^ | 13^ns^ | 26^ns^ | 7^ns^ |
| GH12 | 3 | 3^ns^ | 7^ns^ | 3^ns^ | 4^ns^ | 0^ns^ |
| GH13 | 1921 | 1581^***^ | 1182^***^ | 623^***^ | 1077^***^ | 470^ns^ |
| GH14 | 0 | 0 | 1^ns^ | 0 | 0 | 0 |
| GH15 | 19 | 6^ns^ | 10^ns^ | 41^***^ | 64^***^ | 1^ns^ |
| GH16 | 434 | 341^***^ | 347^ns^ | 71^***^ | 170^***^ | 104^ns^ |
| GH17 | 0 | 0 | 6^ns^ | 0 | 1^ns^ | 0 |
| GH18 | 257 | 232^*^ | 149^ns^ | 172^ns^ | 372^***^ | 34^ns^ |
| GH19 | 17 | 7^ns^ | 10^ns^ | 7^ns^ | 21^ns^ | 4^ns^ |
| GH20 | 480 | 331^***^ | 538^***^ | 144^*^ | 222^**^ | 96^ns^ |
| GH22 | 7 | 2^ns^ | 6^ns^ | 0^ns^ | 1^ns^ | 0^ns^ |
| GH23 | 751 | 629^***^ | 454^ns^ | 333^ns^ | 453^ns^ | 228^ns^ |
| GH24 | 56 | 86^*^ | 79^ns^ | 28^ns^ | 49^ns^ | 14^ns^ |
| GH25 | 717 | 390^***^ | 318^***^ | 61^***^ | 100^***^ | 135^ns^ |
| GH26 | 458 | 217^***^ | 178^***^ | 29^***^ | 90^***^ | 87^ns^ |
| GH27 | 228 | 132^***^ | 154^ns^ | 32^***^ | 53^***^ | 38^ns^ |
| GH28 | 1016 | 356^***^ | 672^ns^ | 88^***^ | 176^***^ | 203^ns^ |
| GH29 | 529 | 390^***^ | 652^***^ | 192^ns^ | 268^***^ | 137^ns^ |
| GH30 | 325 | 174^***^ | 168^ns^ | 42^***^ | 95^***^ | 74^ns^ |
| GH31 | 852 | 451^***^ | 551^ns^ | 147^***^ | 258^***^ | 138^ns^ |
| GH32 | 311 | 289^*^ | 209^ns^ | 63^***^ | 122^***^ | 77^ns^ |
| GH33 | 344 | 261^***^ | 342^ns^ | 147^ns^ | 241^ns^ | 59^ns^ |
| GH35 | 341 | 83^***^ | 166^**^ | 36^***^ | 51^***^ | 71^ns^ |
| GH36 | 421 | 310^***^ | 441^**^ | 88^***^ | 146^***^ | 66^ns^ |
| GH37 | 28 | 32^ns^ | 41^ns^ | 9^ns^ | 10^ns^ | 7^ns^ |
| GH38 | 81 | 120^**^ | 160^***^ | 107^***^ | 158^***^ | 10^ns^ |
| GH39 | 223 | 181^**^ | 332^***^ | 68^ns^ | 102^ns^ | 10^**^ |
| GH42 | 136 | 106^ns^ | 144^ns^ | 85^ns^ | 125^ns^ | 9^ns^ |
| GH43 | 2790 | 1331^***^ | 1249^***^ | 328^***^ | 711^***^ | 557^ns^ |
| GH44 | 16 | 10^ns^ | 7^ns^ | 6^ns^ | 6^ns^ | 2^ns^ |
| GH45 | 28 | 76^***^ | 14^ns^ | 2^ns^ | 7^ns^ | 9^ns^ |
| GH46 | 2 | 3^ns^ | 4^ns^ | 1^ns^ | 2^ns^ | 0^ns^ |
| GH47 | 1 | 0^ns^ | 1^ns^ | 0^ns^ | 0^ns^ | 0^ns^ |
| GH48 | 4 | 2^ns^ | 4^ns^ | 3^ns^ | 15^ns^ | 1^ns^ |
| GH49 | 1 | 0^ns^ | 0^ns^ | 0^ns^ | 0^ns^ | 0^ns^ |
| GH50 | 58 | 48^ns^ | 39^ns^ | 12^ns^ | 28^ns^ | 0^ns^ |
| GH51 | 779 | 280^***^ | 356^***^ | 133^***^ | 234^***^ | 177^ns^ |
| GH52 | 0 | 1^ns^ | 1^ns^ | 5^ns^ | 5^ns^ | 0 |
| GH53 | 432 | 194^***^ | 148^***^ | 24^***^ | 55^***^ | 103^ns^ |
| GH54 | 24 | 32^ns^ | 17^ns^ | 1^ns^ | 1^*^ | 0^ns^ |
| GH55 | 18 | 10^ns^ | 20^ns^ | 3^ns^ | 6^ns^ | 4^ns^ |
| GH57 | 293 | 203^***^ | 158^ns^ | 117^ns^ | 185^ns^ | 90^ns^ |
| GH59 | 16 | 3^ns^ | 4^ns^ | 1^ns^ | 8^ns^ | 6^ns^ |
| GH62 | 0 | 0 | 0^ns^ | 2^ns^ | 3^ns^ | 0 |
| GH63 | 53 | 33^ns^ | 73^ns^ | 18^ns^ | 25^ns^ | 4^ns^ |
| GH64 | 1 | 2^ns^ | 3^ns^ | 1^ns^ | 3^ns^ | 0^ns^ |
| GH65 | 36 | 46^ns^ | 65^*^ | 55^***^ | 119^***^ | 5^ns^ |
| GH66 | 14 | 17^ns^ | 14^ns^ | 9^ns^ | 14^ns^ | 2^ns^ |
| GH67 | 161 | 65^***^ | 56^**^ | 30^**^ | 57^**^ | 26^ns^ |
| GH70 | 0 | 0 | 4^ns^ | 0 | 0 | 0 |
| GH71 | 2 | 0^ns^ | 11^ns^ | 1^ns^ | 0^ns^ | 0^ns^ |
| GH72 | 0 | 0 | 1^ns^ | 0 | 1^ns^ | 0 |
| GH73 | 396 | 295^***^ | 273^ns^ | 134^**^ | 220^ns^ | 94^ns^ |
| GH74 | 121 | 127^ns^ | 100^ns^ | 48^ns^ | 152^**^ | 24^ns^ |
| GH75 | 0 | 1^ns^ | 0 | 0 | 0 | 0 |
| GH76 | 158 | 46^***^ | 48^***^ | 24^***^ | 76^ns^ | 30^ns^ |
| GH77 | 476 | 329^***^ | 317^ns^ | 139^***^ | 185^***^ | 107^ns^ |
| GH78 | 811 | 567^***^ | 767^ns^ | 165^***^ | 287^***^ | 115^ns^ |
| GH79 | 2 | 1^ns^ | 3^ns^ | 0^ns^ | 4^ns^ | 0^ns^ |
| GH80 | 2 | 1^ns^ | 2^ns^ | 0^ns^ | 1^ns^ | 0^ns^ |
| GH81 | 3 | 5^ns^ | 3^ns^ | 5^ns^ | 12^ns^ | 6^ns^ |
| GH82 | 14 | 10^ns^ | 12^ns^ | 2^ns^ | 7^ns^ | 0^ns^ |
| GH84 | 43 | 15^**^ | 45^ns^ | 9^*^ | 20^ns^ | 10^ns^ |
| GH85 | 13 | 0^*^ | 5^ns^ | 2^ns^ | 3^ns^ | 1^ns^ |
| GH87 | 1 | 1^ns^ | 14^ns^ | 3^ns^ | 7^ns^ | 2^ns^ |
| GH88 | 177 | 67^***^ | 86^ns^ | 14^***^ | 40^***^ | 26^ns^ |
| GH89 | 152 | 61^***^ | 90^ns^ | 2^***^ | 6^***^ | 37^ns^ |
| GH91 | 1 | 2^ns^ | 1^ns^ | 2^ns^ | 3^ns^ | 0^ns^ |
| GH92 | 655 | 302^***^ | 403^ns^ | 64^***^ | 131^***^ | 146^ns^ |
| GH93 | 32 | 33^ns^ | 19^ns^ | 9^ns^ | 19^ns^ | 11^ns^ |
| GH94 | 340 | 151^***^ | 200^ns^ | 145^ns^ | 263^ns^ | 40^ns^ |
| GH95 | 486 | 147^***^ | 352^ns^ | 88^***^ | 197^***^ | 102^ns^ |
| GH96 | 0 | 0 | 0 | 1^ns^ | 0 | 0 |
| GH97 | 734 | 315^***^ | 362^***^ | 42^***^ | 79^***^ | 218^ns^ |
| GH98 | 22 | 8^ns^ | 42^ns^ | 3^ns^ | 5^ns^ | 16^ns^ |
| GH99 | 129 | 77^***^ | 178^***^ | 24^*^ | 82^ns^ | 17^ns^ |
| GH100 | 0 | 0 | 0 | 1^ns^ | 0 | 0 |
| GH101 | 0 | 0 | 0 | 0 | 1^ns^ | 0 |
| GH102 | 6 | 12^ns^ | 4^ns^ | 10^ns^ | 7^ns^ | 3^ns^ |
| GH103 | 9 | 13^ns^ | 3^ns^ | 29^***^ | 29^*^ | 4^ns^ |
| GH104 | 0 | 2^ns^ | 0 | 6^**^ | 5^ns^ | 0 |
| GH105 | 623 | 172^***^ | 202^***^ | 40^***^ | 117^***^ | 138^ns^ |
| GH106 | 453 | 184^***^ | 370^ns^ | 85^***^ | 181^***^ | 85^ns^ |
| GH108 | 23 | 21^ns^ | 18^ns^ | 14^ns^ | 23^ns^ | 11^ns^ |
| GH109 | 1505 | 1459^***^ | 1779^ns^ | 1340^ns^ | 2068^***^ | 232^ns^ |
| GH110 | 47 | 32^ns^ | 120^***^ | 15^ns^ | 18^ns^ | 0^ns^ |
| GH112 | 22 | 11^ns^ | 17^ns^ | 16^ns^ | 19^ns^ | 0^ns^ |
| GH113 | 25 | 22^ns^ | 22^ns^ | 7^ns^ | 12^ns^ | 0^ns^ |
| GH114 | 47 | 35^ns^ | 17^ns^ | 4^ns^ | 18^ns^ | 6^ns^ |
| GH115 | 417 | 94^***^ | 168^***^ | 24^***^ | 59^***^ | 83^ns^ |
| GH116 | 20 | 21^ns^ | 54^**^ | 22^ns^ | 41^*^ | 3^ns^ |
| GH117 | 23 | 22^ns^ | 29^ns^ | 7^ns^ | 13^ns^ | 1^ns^ |
| GH119 | 2 | 1^ns^ | 0^ns^ | 0^ns^ | 2^ns^ | 2^ns^ |
| GH120 | 107 | 90^ns^ | 74^ns^ | 47^ns^ | 67^ns^ | 5^ns^ |
| GH121 | 14 | 1^ns^ | 0^ns^ | 3^ns^ | 5^ns^ | 1^ns^ |
| GH123 | 95 | 61^*^ | 138^**^ | 58^ns^ | 87^ns^ | 6^ns^ |
| GH124 | 15 | 8^ns^ | 7^ns^ | 6^ns^ | 14^ns^ | 2^ns^ |
| GH125 | 89 | 58^*^ | 51^ns^ | 23^ns^ | 47^ns^ | 23^ns^ |
| GH126 | 12 | 9^ns^ | 3^ns^ | 8^ns^ | 14^ns^ | 0^ns^ |
| GH127 | 560 | 283^***^ | 423^ns^ | 138^***^ | 225^***^ | 130^ns^ |
| GH128 | 61 | 21^***^ | 34^ns^ | 3^**^ | 10^**^ | 48^ns^ |
| GH129 | 67 | 65^ns^ | 82^ns^ | 33^ns^ | 58^ns^ | 2^ns^ |
| GH130 | 297 | 241^***^ | 207^ns^ | 119^ns^ | 200^ns^ | 56^ns^ |
| GH131 | 0 | 0 | 0 | 1^ns^ | 0 | 0 |
| GH133 | 216 | 136^***^ | 158^ns^ | 47^**^ | 63^***^ | 68^ns^ |
| GH134 | 2 | 0^ns^ | 2^ns^ | 1^ns^ | 0^ns^ | 0^ns^ |
| GH135 | 3 | 1^ns^ | 1^ns^ | 0^ns^ | 3^ns^ | 0^ns^ |
| Total | 31832 | 19465 | 21348 | 7816 | 13787 | 6247 |

Table S2: Counts of carbohydrate binding modules (CBMs) containing proteins predicted in the assembled metagenomes (contigs ≥1000 bp). Statistical significant differences in CAZyme profiles between the camel rumen’s metagenome and the other metagenomes were assessed using Fisher’s exact test. P-values were corrected using FDR method. ns = non-significant, * = FDR-corrected p-value < 0.05, ** = FDR-corrected p-value < 0.01, *** = FDR-corrected p-value < 0.001

| CAZyme family | Camel rumen (this study) | Cow rumen (Hess et al 2011) | Elephant feces (Ilberger et al 2014) | Biogas reactor (Campanaro et at 2016) | Biogas reactor (Gullert et al 2016) | Moose rumen  (Svartström et al 2017) |
| --- | --- | --- | --- | --- | --- | --- |
| CBM1 | 1 | 3^ns^ | 0^ns^ | 0^ns^ | 0^ns^ | 0^ns^ |
| CBM2 | 183 | 69^***^ | 50^***^ | 9^***^ | 42^***^ | 3^***^ |
| CBM3 | 12 | 16^ns^ | 18^ns^ | 12^ns^ | 28^ns^ | 5^ns^ |
| CBM4 | 221 | 261^ns^ | 130^ns^ | 41^***^ | 129^ns^ | 37^ns^ |
| CBM5 | 16 | 39^ns^ | 20^ns^ | 11^ns^ | 34^ns^ | 2^ns^ |
| CBM6 | 177 | 517^ns^ | 152^ns^ | 60^ns^ | 94^ns^ | 37^ns^ |
| CBM8 | 7 | 6^ns^ | 4^ns^ | 1^ns^ | 9^ns^ | 0^ns^ |
| CBM9 | 174 | 256^ns^ | 368^***^ | 142^ns^ | 212^***^ | 20^ns^ |
| CBM10 | 0 | 0 | 1^ns^ | 1^ns^ | 0 | 0 |
| CBM11 | 13 | 34^ns^ | 17^ns^ | 9^ns^ | 11^ns^ | 7^ns^ |
| CBM12 | 61 | 160^ns^ | 79^ns^ | 23^ns^ | 52^ns^ | 7^ns^ |
| CBM13 | 171 | 143^ns^ | 82^ns^ | 21^***^ | 41^***^ | 59^ns^ |
| CBM14 | 4 | 2^ns^ | 1^ns^ | 1^ns^ | 0^ns^ | 0^ns^ |
| CBM15 | 0 | 1^ns^ | 2^ns^ | 1^ns^ | 2^ns^ | 2^ns^ |
| CBM16 | 131 | 218^ns^ | 133^ns^ | 112^ns^ | 142^ns^ | 13^ns^ |
| CBM17 | 0 | 0 | 2^ns^ | 3^ns^ | 4^ns^ | 0 |
| CBM18 | 1 | 1^ns^ | 0^ns^ | 1^ns^ | 1^ns^ | 0^ns^ |
| CBM19 | 6 | 9^ns^ | 1^ns^ | 0^ns^ | 1^ns^ | 1^ns^ |
| CBM20 | 159 | 109^**^ | 70^ns^ | 16^***^ | 29^***^ | 37^ns^ |
| CBM21 | 2 | 3^ns^ | 2^ns^ | 0^ns^ | 5^ns^ | 0^ns^ |
| CBM22 | 94 | 90^ns^ | 66^ns^ | 42^ns^ | 61^ns^ | 8^ns^ |
| CBM23 | 23 | 49^ns^ | 20^ns^ | 20^ns^ | 27^ns^ | 3^ns^ |
| CBM24 | 1 | 1^ns^ | 2^ns^ | 0^ns^ | 0^ns^ | 0^ns^ |
| CBM25 | 13 | 10^ns^ | 1^ns^ | 52^***^ | 62^***^ | 5^ns^ |
| CBM26 | 118 | 158^ns^ | 72^ns^ | 5^***^ | 46^ns^ | 78^ns^ |
| CBM27 | 10 | 6^ns^ | 6^ns^ | 1^ns^ | 5^ns^ | 0^ns^ |
| CBM28 | 1 | 1^ns^ | 2^ns^ | 1^ns^ | 1^ns^ | 0^ns^ |
| CBM29 | 6 | 3^ns^ | 4^ns^ | 1^ns^ | 1^ns^ | 1^ns^ |
| CBM30 | 25 | 46^ns^ | 22^ns^ | 6^ns^ | 3^ns^ | 1^ns^ |
| CBM31 | 4 | 5^ns^ | 6^ns^ | 4^ns^ | 3^ns^ | 2^ns^ |
| CBM32 | 686 | 707^*^ | 445^ns^ | 177^***^ | 400^ns^ | 94^ns^ |
| CBM33 | 0 | 0 | 0 | 0 | 0 | 0 |
| CBM34 | 37 | 48^ns^ | 19^ns^ | 16^ns^ | 16^ns^ | 2^ns^ |
| CBM35 | 280 | 411^ns^ | 208^ns^ | 77^ns^ | 190^ns^ | 58^ns^ |
| CBM36 | 11 | 13^ns^ | 7^ns^ | 1^ns^ | 7^ns^ | 1^ns^ |
| CBM37 | 507 | 455^***^ | 225^***^ | 29^***^ | 92^***^ | 45^**^ |
| CBM38 | 15 | 34^ns^ | 11^ns^ | 5^ns^ | 20^ns^ | 0^ns^ |
| CBM39 | 16 | 9^ns^ | 9^ns^ | 1^ns^ | 2^ns^ | 1^ns^ |
| CBM40 | 160 | 140^ns^ | 90^ns^ | 62^ns^ | 124^ns^ | 16^ns^ |
| CBM41 | 12 | 33^ns^ | 10^ns^ | 13^ns^ | 28^ns^ | 0^ns^ |
| CBM42 | 5 | 2^ns^ | 2^ns^ | 2^ns^ | 5^ns^ | 1^ns^ |
| CBM43 | 1 | 3^ns^ | 5^ns^ | 2^ns^ | 0^ns^ | 0^ns^ |
| CBM44 | 343 | 369^ns^ | 116^***^ | 139^ns^ | 308^ns^ | 57^ns^ |
| CBM45 | 1 | 1^ns^ | 0^ns^ | 0^ns^ | 0^ns^ | 0^ns^ |
| CBM46 | 38 | 34^ns^ | 28^ns^ | 14^ns^ | 22^ns^ | 2^ns^ |
| CBM47 | 6 | 6^ns^ | 13^ns^ | 4^ns^ | 2^ns^ | 0^ns^ |
| CBM48 | 201 | 326^ns^ | 147^ns^ | 59^ns^ | 95^ns^ | 12^*^ |
| CBM49 | 5 | 8^ns^ | 1^ns^ | 3^ns^ | 2^ns^ | 2^ns^ |
| CBM50 | 1111 | 984^***^ | 744^ns^ | 856^ns^ | 1493^***^ | 235^ns^ |
| CBM51 | 60 | 107^ns^ | 58^ns^ | 20^ns^ | 41^ns^ | 24^ns^ |
| CBM52 | 2 | 10^ns^ | 2^ns^ | 0^ns^ | 1^ns^ | 1^ns^ |
| CBM53 | 1 | 5^ns^ | 5^ns^ | 0^ns^ | 4^ns^ | 1^ns^ |
| CBM54 | 24 | 37^ns^ | 26^ns^ | 51^***^ | 120^***^ | 2^ns^ |
| CBM55 | 1 | 3^ns^ | 4^ns^ | 1^ns^ | 1^ns^ | 1^ns^ |
| CBM56 | 431 | 321^***^ | 190^***^ | 34^***^ | 64^***^ | 87^ns^ |
| CBM57 | 18 | 19^ns^ | 17^ns^ | 2^ns^ | 12^ns^ | 4^ns^ |
| CBM58 | 7 | 9^ns^ | 2^ns^ | 2^ns^ | 3^ns^ | 2^ns^ |
| CBM59 | 16 | 12^ns^ | 5^ns^ | 4^ns^ | 7^ns^ | 2^ns^ |
| CBM60 | 1 | 1^ns^ | 5^ns^ | 0^ns^ | 1^ns^ | 1^ns^ |
| CBM61 | 464 | 154^***^ | 164^***^ | 11^***^ | 49^***^ | 179^ns^ |
| CBM62 | 94 | 69^ns^ | 77^ns^ | 14^*^ | 42^ns^ | 20^ns^ |
| CBM63 | 20 | 32^ns^ | 11^ns^ | 5^ns^ | 9^ns^ | 6^ns^ |
| CBM64 | 1 | 4^ns^ | 3^ns^ | 2^ns^ | 5^ns^ | 0^ns^ |
| CBM65 | 6 | 2^ns^ | 5^ns^ | 1^ns^ | 4^ns^ | 2^ns^ |
| CBM66 | 176 | 146^*^ | 123^ns^ | 99^ns^ | 174^ns^ | 30^ns^ |
| CBM67 | 324 | 344^ns^ | 219^ns^ | 57^***^ | 95^***^ | 39^ns^ |
| CBM68 | 2 | 4^ns^ | 6^ns^ | 3^ns^ | 2^ns^ | 0^ns^ |
| CBM69 | 28 | 27^ns^ | 23^ns^ | 1^ns^ | 7^ns^ | 11^ns^ |
| CBM70 | 10 | 14^ns^ | 22^ns^ | 11^ns^ | 8^ns^ | 0^ns^ |
| CBM71 | 3 | 0^ns^ | 0^ns^ | 0^ns^ | 1^ns^ | 0^ns^ |
| CBM72 | 68 | 22^***^ | 14^**^ | 3^***^ | 3^***^ | 14^ns^ |
| CBM73 | 13 | 7^ns^ | 8^ns^ | 0^ns^ | 1^ns^ | 0^ns^ |
| CBM74 | 2 | 9^ns^ | 0^ns^ | 0^ns^ | 9^ns^ | 0^ns^ |
| CBM75 | 1 | 1^ns^ | 1^ns^ | 0^ns^ | 0^ns^ | 1^ns^ |
| CBM76 | 8 | 3^ns^ | 4^ns^ | 0^ns^ | 4^ns^ | 3^ns^ |
| CBM77 | 27 | 4^**^ | 23^ns^ | 2^ns^ | 7^ns^ | 12^ns^ |
| CBM78 | 34 | 26^ns^ | 8^ns^ | 2^ns^ | 2^**^ | 2^ns^ |
| CBM79 | 5 | 2^ns^ | 8^ns^ | 0^ns^ | 3^ns^ | 2^ns^ |
| CBM80 | 4 | 5^ns^ | 3^ns^ | 1^ns^ | 0^ns^ | 1^ns^ |
| Total | 6920 | 7198 | 4449 | 2382 | 4530 | 1301 |

Table S3: The predicted carbohydrate esterases (CEs) characterized in the assembled metagenomes (contigs ≥1000 bp). Statistical significant differences in CAZyme profiles between the camel rumen’s metagenome and the other metagenomes were assessed using Fisher’s exact test. P-values were corrected using FDR method. ns = non-significant, * = FDR-corrected p-value < 0.05, ** = FDR-corrected p-value < 0.01, *** = FDR-corrected p-value < 0.001

| CAZyme family | Camel rumen (this study) | Cow rumen (Hess et al 2011) | Elephant feces (Ilberger et al 2014) | Biogas reactor (Campanaro et at 2016) | Biogas reactor (Gullert et al 2016) | Moose rumen  (Svartström et al 2017) |
| --- | --- | --- | --- | --- | --- | --- |
| CE1 | 2885 | 2399^***^ | 1699^***^ | 595^***^ | 1085^***^ | 447^ns^ |
| CE2 | 463 | 391^***^ | 326^ns^ | 46^***^ | 60^***^ | 77^ns^ |
| CE3 | 744 | 582^***^ | 710^ns^ | 221^***^ | 321^***^ | 110^ns^ |
| CE4 | 928 | 877^***^ | 794^ns^ | 601^ns^ | 1113^***^ | 141^ns^ |
| CE5 | 29 | 11^ns^ | 25^ns^ | 16^ns^ | 19^ns^ | 6^ns^ |
| CE6 | 644 | 391^***^ | 378^ns^ | 76^***^ | 163^***^ | 100^ns^ |
| CE7 | 509 | 271^***^ | 365^ns^ | 138^***^ | 206^***^ | 103^ns^ |
| CE8 | 602 | 132^***^ | 167^***^ | 35^***^ | 108^***^ | 125^ns^ |
| CE9 | 169 | 219^ns^ | 309^***^ | 215^***^ | 256^***^ | 24^ns^ |
| CE10 | 2758 | 1468^***^ | 1282^***^ | 450^***^ | 914^***^ | 392^*^ |
| CE11 | 247 | 157^***^ | 149^ns^ | 61^*^ | 78^***^ | 80^ns^ |
| CE12 | 725 | 340^***^ | 309^***^ | 71^***^ | 162^***^ | 156^ns^ |
| CE13 | 17 | 18^ns^ | 29^ns^ | 2^ns^ | 1^ns^ | 0^ns^ |
| CE14 | 243 | 283^ns^ | 325^***^ | 159^ns^ | 312^***^ | 45^ns^ |
| CE15 | 345 | 201^***^ | 230^ns^ | 89^**^ | 136^***^ | 40^ns^ |
| Total | 11308 | 7740 | 7097 | 2775 | 4934 | 1846 |

Table S4: Table shows the auxiliary activity domain containing proteins (AAs) identified in the assembled metagenomes (contigs ≥1000 bp).Statistical significant differences in CAZyme profiles between the camel rumen’s metagenome and the other metagenomes were assessed using Fisher’s exact test. P-values were corrected using FDR method. ns = non-significant, * = FDR-corrected p-value  0.05, ** = FDR-corrected p-value  0.01, *** = FDR-corrected p-value  0.001

| CAZymes family | Camel rumen (this study) | Cow rumen (Hess et al 2011) | Elephant feces (Ilberger et al 2014) | Biogas reactor (Campanaro et at 2016) | Biogas reactor (Gullert et al 2016) | Moose rumen  (Svartström et al 2017) |
| --- | --- | --- | --- | --- | --- | --- |
| AA1 | 0 | 0 | 0 | 1^ns^ | 0 | 0 |
| AA2 | 0 | 0 | 1^ns^ | 5^ns^ | 11^ns^ | 0 |
| AA3 | 38 | 27^ns^ | 18^ns^ | 54^ns^ | 79^***^ | 9^ns^ |
| AA4 | 40 | 17^ns^ | 57^ns^ | 96^ns^ | 109^***^ | 4^ns^ |
| AA6 | 1055 | S^***^ | 365^***^ | 444^ns^ | 616^ns^ | 213^ns^ |
| AA7 | 29 | 17^ns^ | 36^ns^ | 30^ns^ | 61^**^ | 5^ns^ |
| AA9 | 0 | 0 | 1^ns^ | 0 | 0 | 0 |
| AA10 | 1 | 3^ns^ | 0 | 0^ns^ | 1^ns^ | 0^ns^ |
| AA11 | 0 | 4^ns^ | 0 | 0 | 1^ns^ | 0 |
| AA12 | 0 | 0 | 0 | 5^ns^ | 4^ns^ | 0 |
| AA13 | 1 | 0^ns^ | 0^ns^ | 0^ns^ | 0 | 0^ns^ |
| Total | 1164 | 920 | 478 | 635 | 882 | 231 |

Table S5: Table presents the distribution of dockerin, cohesion, and surface layer homology (SLH) domain containing proteins predicted in the assembled metagenomes (contigs ≥1000 bp).Statistical significant differences in CAZyme profiles between the camel rumen’s metagenome and the other metagenomes were assessed using Fisher’s exact test. P-values were corrected using FDR method. ns = non-significant, * = FDR-corrected p-value  0.05, ** = FDR-corrected p-value  0.01, *** = FDR-corrected p-value  0.001

| CAZyme family | Camel rumen (this study) | Cow rumen (Hess et al 2011) | Elephant feces (Ilberger et al 2014) | Biogas reactor (Campanaro et at 2016) | Biogas reactor (Gullert et al 2016) | Moose rumen  (Svartström et al 2017) |
| --- | --- | --- | --- | --- | --- | --- |
| Dockerin | 1161 | 481^***^ | 316^***^ | 74^***^ | 168^***^ | 378^ns^ |
| Cohesin | 146 | 94^***^ | 135^ns^ | 73^ns^ | 152^ns^ | 23^ns^ |
| SLH | 578 | 574^**^ | 536^ns^ | 592^***^ | 1213^***^ | 37^***^ |
| SusD | 1684 | 880^***^ | 690^**^ | 124^***^ | 405^***^ | 441^ns^ |
| SusD-like | 94 | 32^***^ | 24^**^ | 8^***^ | 15^**^ | 16^ns^ |
| SusD-like_2 | 136 | 64^***^ | 81^**^ | 30^***^ | 43^***^ | 40^ns^ |
| SusD-like_3 | 1804 | 770^***^ | 847^***^ | 153^***^ | 436^**^ | 446^ns^ |
| SusC | 4650 | 1800^***^ | 1976^***^ | 557^***^ | 1117^***^ | 1342^ns^ |
| Total | 10253 | 4695 | 4605 | 1611 | 3549 | 2723 |

Table S6: The numbers of predicted polysaccharide lyases (PLs) detected in the assembled metagenomes (contigs ≥1000 bp).Statistical significant differences in CAZyme profiles between the camel rumen’s metagenome and the other metagenomes were assessed using Fisher’s exact test. P-values were corrected using FDR method. ns = non-significant, * = FDR-corrected p-value  0.05, ** = FDR-corrected p-value  0.01, *** = FDR-corrected p-value  0.001

| CAZyme family | Camel rumen (this study) | Cow rumen (Hess et al 2011) | Elephant feces (Ilberger et al 2014) | Biogas reactor (Campanaro et at 2016) | Biogas reactor (Gullert et al 2016) | Moose rumen  (Svartström et al 2017) |
| --- | --- | --- | --- | --- | --- | --- |
| PL1 | 631 | 171^***^ | 215^***^ | 24^***^ | 112^***^ | 185^ns^ |
| PL2 | 0 | 0 | 0 | 2^ns^ | 5^ns^ | 0 |
| PL3 | 2 | 3^ns^ | 2^ns^ | 2^ns^ | 1^ns^ | 1^ns^ |
| PL4 | 8 | 2^ns^ | 3^ns^ | 2^ns^ | 8^ns^ | 9^ns^ |
| PL5 | 9 | 2^ns^ | 5^ns^ | 2^ns^ | 1^ns^ | 0^ns^ |
| PL6 | 20 | 13^ns^ | 43^*^ | 14^ns^ | 28^ns^ | 8^ns^ |
| PL7 | 4 | 2^ns^ | 2^ns^ | 0^ns^ | 0^ns^ | 3^ns^ |
| PL8 | 19 | 4^ns^ | 46^**^ | 6^ns^ | 13^ns^ | 6^ns^ |
| PL9 | 245 | 80^***^ | 171^ns^ | 63^*^ | 127^ns^ | 41^ns^ |
| PL10 | 106 | 24^***^ | 28^***^ | 9^***^ | 23^***^ | 33^ns^ |
| PL11 | 421 | 76^***^ | 112^***^ | 21^***^ | 67^***^ | 130^ns^ |
| PL12 | 173 | 149^ns^ | 151^ns^ | 40^ns^ | 112^ns^ | 20^ns^ |
| PL13 | 0 | 0 | 0 | 0 | 0 | 0 |
| PL14 | 6 | 18^ns^ | 7^ns^ | 2^ns^ | 4^ns^ | 0^ns^ |
| PL15 | 20 | 31^ns^ | 7^ns^ | 13^ns^ | 37^ns^ | 1^ns^ |
| PL16 | 0 | 0 | 0 | 0 | 0 | 0 |
| PL17 | 19 | 25^ns^ | 17^ns^ | 18^ns^ | 62^***^ | 3^ns^ |
| PL18 | 1 | 7^ns^ | 3^ns^ | 2^ns^ | 5^ns^ | 0^ns^ |
| PL19 | 0 | 0 | 0 | 0 | 0 | 0 |
| PL20 | 2 | 4^ns^ | 1^ns^ | 0^ns^ | 2^ns^ | 0^ns^ |
| PL21 | 7 | 25^ns^ | 16^ns^ | 4^ns^ | 5^ns^ | 0^ns^ |
| PL22 | 181 | 106^***^ | 66^**^ | 40^*^ | 64^**^ | 35^ns^ |
| PL23 | 0 | 0 | 0 | 0 | 0 | 0 |
| PL24 | 1 | 0^ns^ | 2^ns^ | 0^ns^ | 1^ns^ | 1^ns^ |
| Total | 1875 | 742 | 897 | 264 | 677 | 476 |
